# Redescription of *Tintinnopsis everta* Kofoid and Campbell 1929 (Alveolata, Ciliophora, Tintinnina) Based on Taxonomic and Genetic Analyses—Discovery of a New Complex Ciliary Pattern

**DOI:** 10.1111/jeu.12496

**Published:** 2018-01-31

**Authors:** Michael S. Gruber, Michaela Strüder‐Kypke, Sabine Agatha

**Affiliations:** ^1^ Department of Biosciences University of Salzburg 5020 Salzburg Austria; ^2^ Department of Molecular and Cellular Biology University of Guelph Guelph ON N1G 2W1 Canada

**Keywords:** Barcoding, cell division, lorica, marine plankton, morphology, phylogeny

## Abstract

The about 1,000 species of tintinnid ciliates are identified and classified almost exclusively based on their lorica features, although the shortcomings of this structure are well‐known, e.g. causing uncertain species limitations and nonmonophyletic taxa. Hence, the present redescription of *Tintinnopsis everta* Kofoid and Campbell, 1929 considers not only the lorica characteristics, but focuses on cell and genetic features. The species is redescribed from the North Atlantic and adjacent sea areas, namely the east coast of the USA, using live observation, protargol‐stained material, scanning electron microscopy, and genetic analyses. The main stages of cell division are described, and the species’ phylogenetic relationships are inferred from morphological data and the small subunit ribosomal RNA gene sequence. The estimates of its biogeographical distribution and autecology are based on a literature survey. The species is characterised by a complex somatic ciliary pattern with a unique position of the posterior kinety and a conspicuously large distance between the somatic ciliary fields and the collar membranelles. The phylogenetic relationships of *Tintinnopsis everta* vary in the molecular trees depending on the algorithms used and are, therefore, regarded as unresolved. Nevertheless, the new kind of complex somatic ciliary pattern distinctly contributes to a better understanding of the tintinnid biodiversity and evolution and provides features for a future split of the nonmonophyletic genus *Tintinnopsis*.

THE first tintinnid was described by Müller (1779) under the name *Trichoda inquilinus*; hence, it was affiliated with totally different kinds of ciliates. In 1803, the new genus *Tintinnus* was established for this species (Schrank 1803). Claparède and Lachmann (1858) extended and revised *Tintinnus* to contain 17 species known at that time and provided some information on the morphology and ecology of the taxa. Nine years later, the genus *Tintinnopsis* was erected by Stein (1867) with *Tintinnopsis beroidea* as type for species with an agglutinated and hard lorica.

While detailed live observations and staining procedures were applied since the 1930th to reveal cytological characters in aloricate ciliates, the comparatively robust loricae, that better withstand rough sampling procedures and diverse preservation methods, provided taxonomic features easier and faster accessible than those of the fragile cells in tintinnids. During the past 150 years, a huge body of literature accumulated describing the diversity and distribution of tintinnid ciliates identified by lorica features (Santoferrara et al. 2016). The lorica remained the sole structure for identification of the about 1,000 tintinnid species till today, although the taxonomic shortcomings of the lorica features were already discussed by Hofker (1931). Beyond comprehensive observations on field material (Davis 1981; Kofoid and Campbell 1929, 1939; Laval‐Peuto 1994), clear evidence for the ability to construct different types of loricae (polymorphism) was provided by laboratory cultures (Laval‐Peuto 1977, 1981) and barcoding (Santoferrara et al. 2015).

Hence, the current lorica‐based taxonomy apparently overestimates tintinnid diversity. On the other hand, molecular approaches indicate the existence of cryptic species in at least some taxa; the separation of these species with identical or very similar loricae will increase the number of recognised tintinnid species again. Independent of their total diversity, which can hardly be estimated today, the limitations of a lorica‐based taxonomy and classification are obvious and particularly impressive in the nonmonophyly of the genus *Tintinnopsis*. A revision, however, requires the application of modern investigation techniques (detailed live observation, staining methods, electron microscopy, and DNA sequencing), especially, in the type species *T. beroidea* Stein, 1867. Santoferrara et al. (2017) revised the phylogenetic relationships and established eleven “Tintinnida” clades all including *Tintinnopsis* species besides *incertae sedis* genera with sparsely agglutinated (*Leprotintinnus*,* Rhizodomus*,* Stylicauda*) and hyaline (*Climacocylis*,* Helicostomella*) loricae (Santoferrara et al. 2017). In the future, with a more comprehensive knowledge about tintinnid morphology, specifically cell features, these monophyletic clades will receive a systematic rank. Therefore, the genera *Rhizodomus* and *Stylicauda* are maintained here and in Agatha and Strüder‐Kypke (2013), although some authors discuss synonymisation with the genus *Tintinnopsis* (da Cunha and da Fonseca 1917; Laval‐Peuto 1994).

Morphology is still the key to the old literatures, despite worldwide environmental high‐throughput sequencing gathered an enormous amount of distribution data for unidentified OTUs of eukaryotic marine plankton organisms including a diverse ciliate community over the last five years (Gimmler et al. 2016; de Vargas et al. 2015). Generally, the short sequences can only be assigned to higher taxonomic levels; unequivocal species identification usually necessitates longer sequences and 100% similarity to a sequence of a properly determined species deposited in GenBank. Currently, however, small subunit ribosomal DNA sequences as reference are only available for about 10% of the known species (Warren et al. 2017). To overcome this problem, collaborations combining morphological, molecular, and ecological investigation techniques are essential, providing DNA barcodes for reliably identified species. This will definitely increase the quality of future phylogenetic studies and ecological surveys, which are increasingly based on environmental sequencing (Santoferrara et al. 2017).

Among the cytological features, the ciliary pattern is crucial as in other ciliates (Warren et al. 2017). Yet, the preliminary cytological data cover only about 3% of the more than 1,000 tintinnid species (see review by Agatha and Strüder‐Kypke 2013). Agatha and Strüder‐Kypke (2007) predicted the discovery of not only further somatic ciliary patterns, but also of small differences within the known patterns, both providing relevant features for revising the tintinnid taxonomy and classification in combination with genetic analyses.

In the present paper, a species of the non‐monophyletic genus *Tintinnopsis*, namely, *Tintinnopsis everta* Kofoid and Campbell, 1929; was redescribed, integrating morphological and molecular features and inferring its phylogenetic relationships following the recommendations and protocols published by Santoferrara et al. (2016).

## Materials and Methods

### Collection

All samples were taken from surface waters by horizontal towing a 10‐μm meshed plankton net at different sites along the east coast of the USA. Salinity measurements were performed with a refractometer and temperature measurements with a temperature probe. The specimens were collected from (i) the Indian River near the Smithsonian Marine Station in Fort Pierce, Florida (27°41′32″N, 80°23′17″W) on 4th August 2010 at a water temperature of 29 °C and a salinity of 30‰, (ii) the inlet in Ocean City at the Atlantic coast of Maryland (38°19′53″N, 75°05′32″W) on 11th August 2010 at a water temperature of 21 °C and a salinity of 30‰, and (iii) the Chesapeake Bay in Maryland (37°44′N, 76°11′W) on 14th June 1991 at a water temperature of 25 °C and salinities of about 25‰.

### Taxonomic studies

Live observation was performed on specimens from the Indian River and Ocean City. Cell movement was studied in a Petri dish (about 5 cm across, water depth about 0.8 cm) under a dissecting microscope at about 22–25 °C. Morphology of the living cell was investigated under compound microscopes (Zeiss Axioscope, Carl Zeiss Inc., Thornwood, NY) equipped with a high‐power oil immersion objective as well as bright‐field and interference contrast optics. The microscopes were equipped with a Nikon E5000 camera in the Smithsonian Marine Station (Florida) and a Zeiss Axiocam in the Smithsonian Environmental Research Center (Maryland).

Cells from the Chesapeake Bay were preserved in a modified Bouin's fixative (Coats and Heinbokel 1982) and stained, following the QPS method (Quantitative Protargol Stain; Montagnes and Lynn 1987). Morphology was investigated under an Olympus BX51 compound microscope equipped with a high‐power oil immersion objective, bright‐field and interference contrast optics, and a Canon EOS 7D digital camera. For scanning electron microscopy (SEM), cells from Ocean City were fixed for 30 min in a modified Parducz’ solution made of six parts of 2% osmium tetroxide (OsO_4_, w/v) in artificial sea water and one part of saturated aqueous mercuric‐chloride (HgCl_2_; Valbonesi and Luporini 1990); further steps were according to Foissner (1991). Counts and measurements on protargol‐stained cells were performed at 1,200× magnification, in vivo measurements were made at 40–1,200× magnification.

### Illustrations

The drawing of the live specimen combines data from live observation, protargol staining, and scanning electron microscopy (SEM), i.e. hand sketches of material collected in the Indian River and the inlet of Ocean City and mean measurements of live and preserved specimens. The loricae are often slightly deformed and comparatively indistinct in protargol slides, but perfectly fit those of specimens studied in vivo and scanning electron micrographs; hence, the lorica data are from the latter specimens. The line drawings of protargol‐stained cells were made with a camera lucida. The kinetal map depicts the ciliary pattern of a protargol‐stained morphostatic specimen in two dimensions, following Agatha and Riedel‐Lorjé (2006). Kineties are drawn to extend longitudinally from their (anterior) starting point, neglecting their curvatures, except for the ventral kinety and the last kinety of the lateral ciliary field, whose courses might be of taxonomic significance. Cilia were only drawn in the posterior and dorsal kineties, in which the anterior dikinetidal basal bodies are unciliated; otherwise, all basal bodies have associated a cilium.

### Terminology

Generally, terminology follows Agatha and Riedel‐Lorjé (2006), but two terms have to be refined for all tintinnids. Dorsal kinety/ies: one or more kineties on dorsal side that are separated from the right and left ciliary fields by distinct blank stripes. They are leftwards curved and usually the longest kineties, extending from the membranellar zone to the base of the peduncle. Posterior kinety: a distinctly anteriorly shortened and rightwards curved kinety, whose anterior end is near the lower margin of the left or lateral ciliary field, while its posterior portion runs somewhat parallel to the dorsal kinety; hence, posterior and dorsal kinety diverge in their anterior portions. Both kineties are composed of dikinetids having a cilium associated only with the posterior basal body; reports of a monokinetidal structure might result from insufficient staining of the unciliated basal bodies, but verification by transmission electron microscopy (TEM) is pending.

### DNA extraction and sequencing

After detailed live observations at high magnification (up to 1,200×), specimens matching in similar‐sized loricae with a flared and annulated collar, a conspicuous distance between the collar membranelles and the ciliary fields, and an extension of the undisturbed cells far beyond the lorica rim were picked from material sampled in the Indian River and preserved in 80% ethanol; no similar tintinnid species that could have been confused occurred.

The DNA was extracted from the cells, using the DNEasy Blood and Tissue kit (Qiagen, Mississauga, ON, Canada) according to the manufacturer's protocol, with the exception that cells were lysed for 30 min and only 100 μl of buffer AE was used for elution. Amplification of the small subunit ribosomal RNA (SSU rRNA) gene with primers 300F (5′‐AGGGTTCGATTCCGGAG‐3′; Elwood et al. 1985) and Reverse B (5′‐TGATCCTTCTGCAGGTTCACCTAC‐3′; Medlin et al. 1988) followed a standard PCR protocol, and the amplified product was purified with the MinElute Gel purification kit (Qiagen). Finally, the SSU rRNA gene was sequenced in both directions with a 3730 DNA Analyzer (Applied Biosystems, Burlington, ON), using the amplification primers plus two internal primers (690F and 690R; Elwood et al. 1985).

### Sequence analysis and alignment

The sequence fragments were assembled into contigs with Sequencher ver. 5.4 (Gene Codes Corp., Ann Arbor, MI, USA), trimmed at the ends, and checked for sequencing errors. *Tintinnopsis* SSU rRNA gene sequences as well as selected other tintinnid sequences were aligned in MEGA ver. 6.06 (Tamura et al. 2013), using the MUSCLE algorithm (Edgar 2004) and subsequent manual refinement. Additional choreotrichid and oligotrichid sequences were used as outgroup. For GenBank accession numbers, see Table S1 in the supplementary material. Distance data were inferred from the sequence alignment with only the ends trimmed. Pairwise distances were calculated with MEGA ver. 6 based on the Kimura‐2‐Parameter model (Kimura 1980).

### Phylogenetic analyses

The SSU rRNA gene alignment was imported into Gblocks ver. 0.91b (Castresana 2000), and ambiguously aligned, hypervariable regions were removed from the data sets. The final alignment for phylogenetic analyses comprised 1,690 nucleotides (93% of original alignment). The best model for nucleotide substitution in the dataset was calculated by jModeltest ver. 2.1.3 (Darriba et al. 2012; Guindon and Gascuel 2003) on the CIPRES Science Gateway (Miller et al. 2010). Under the AIC criterion, the General Time Reversible (GTR) Model with gamma distribution (Γ) and proportion of invariable sites (I) was selected.

Four standard phylogenetic analyses were performed: Maximum Likelihood (ML), Bayesian Inference (BI), Maximum Parsimony (MP), and Neighbor Joining (NJ). The ML and BI analyses were performed through the CIPRES Science Gateway (Miller et al. 2010). The ML analysis was run with RAxML‐HPC2 on XSEDE (Stamatakis et al. 2008) with 1,000 rapid bootstrap replicates and a subsequent thorough ML search, using the GTR + I + Γ model. Bayesian Inference was computed with MrBayes ver. 3.2.2. on XSEDE (Ronquist and Huelsenbeck 2003), also using the GTR + I + Γ model. Two parallel runs were performed. The maximum posterior probability of a phylogeny out of 5,000,000 generations, respectively, approximating it with the Markov chain Monte Carlo and sampling every 200th generation was calculated, discarding the first 25% of trees as burn‐in. Average standard deviation of split frequencies (< 0.01) was used to assess convergence of the two runs. The PAUP analysis (PAUP ver. 4.0a150 for Macintosh; Swofford 2002) determined 388 parsimony‐informative characters. Species were added stepwise and randomly, the tree bisection‐reconnection branch‐swapping algorithm was used, and the data were bootstrapped 1,000 times. PHYLIP ver. 3.695 (Felsenstein 2009) was employed to calculate genetic distances with the Kimura‐2‐Parameter model (Kimura 1980), using DNADIST. The distance trees were constructed with NEIGHBOR, using the Neighbor Joining algorithm (Saitou and Nei 1987). The data were bootstrapped 1,000 times.

## Results

***Tintinnopsis everta*** **Kofoid and Campbell,** **1929**

### Remarks

The specimens collected at the three sampling sites match in lorica shape, size, number of collar annuli, a comparatively high transparency of the lorica wall, an extraordinary extension of the undisturbed living cell far beyond the lorica rim, and especially, a uniquely large distance between the membranellar zone and the ciliary fields; hence, conspecificity is beyond doubt and the data are lumped.

### Redescription

**Figure 1:**
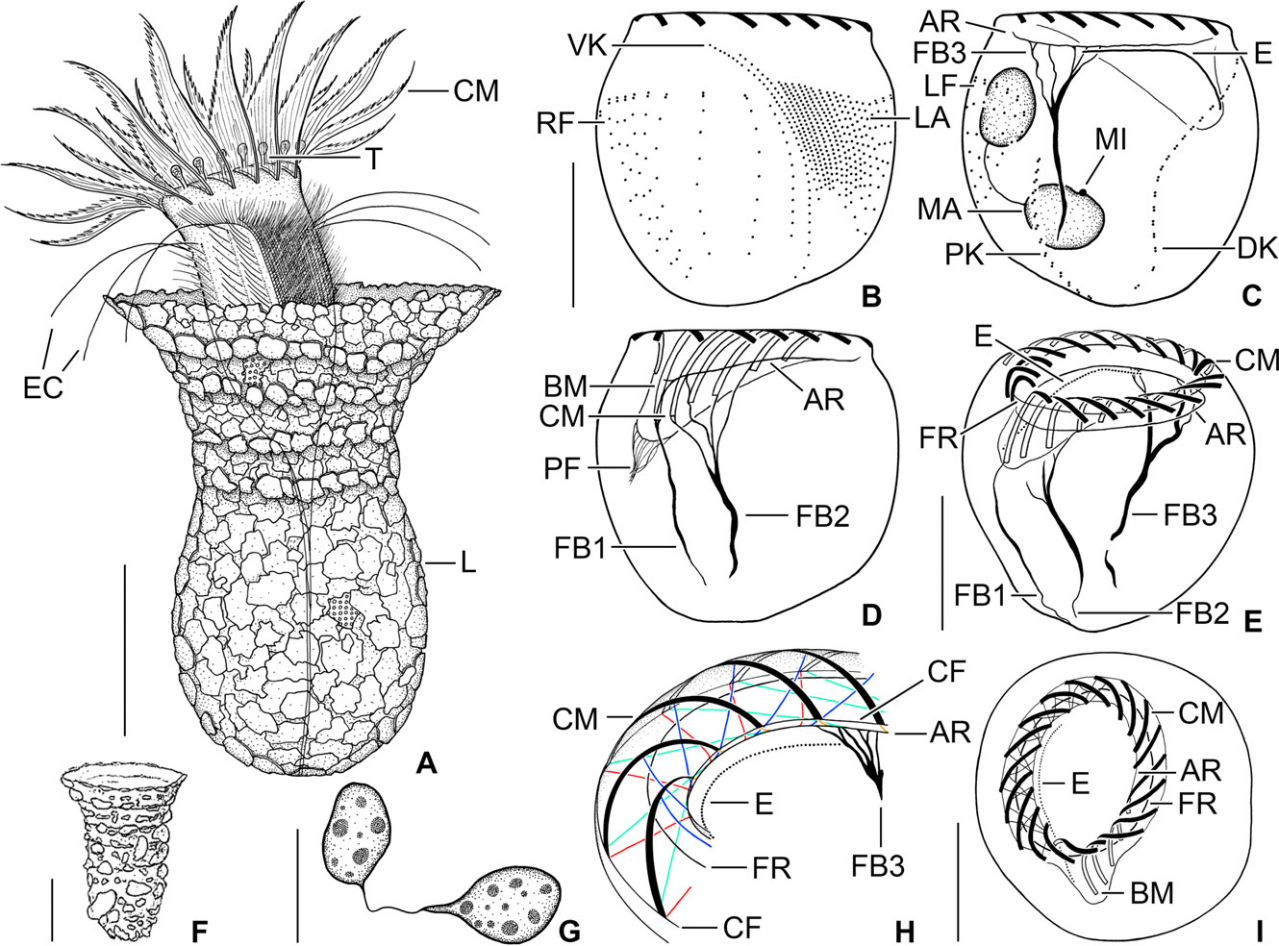
*Tintinnopsis everta*, specimens from the east coast of the USA (**A–E**,** G–I**) and Baltic Sea type specimen (**F**) from life (A, F) and after protargol staining (B–E, G–I). (A) Representative specimen. (B, C) Ciliary pattern of ventral and dorsal sides of same specimen. (D) Ventral view showing the buccal cavity and fibre bundles associated with the elongated collar membranelles and the buccal membranelle. (E, I) Oblique top views showing the membranellar zone and the subjacent fibre system. (F) Lateral view of lorica (from Laackmann 1908). (G) Macronucleus nodules. (H) Schematic illustration of the complex adoral system of argyrophilic structures/fibres. AR, adoral ring; BM, buccal membranelle; CF, circular fibres; CM, collar membranelles; DK, dorsal kinety; E, endoral membrane; EC, elongated cilia; FB1, fibre bundle of buccal membranelle; FB2, fibre bundle of elongated collar membranelles; FB3, fibre bundle originating in dorsal portion of adoral ring; FR, fibrillar ring; L, lorica; LA, lateral ciliary field; LF, left ciliary field; MA, macronucleus nodule; MI, micronucleus; PF, pharyngeal fibres; PK, posterior kinety; RF, right ciliary field; T, tentaculoids; VK, ventral kinety. Scale bars = 30 μm (A, F), 15 μm (B–E, H), 10 μm (G).

**Figure 2:**
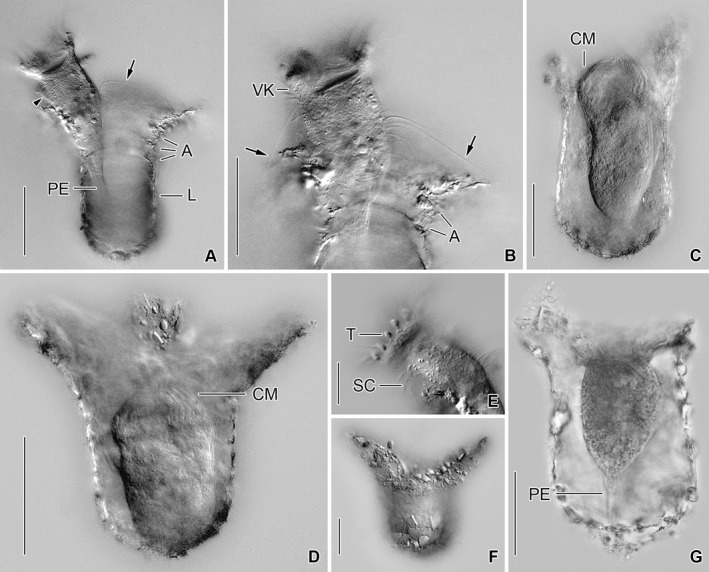
*Tintinnopsis everta*, specimens from the inlet in Ocean City from life (**A–F**) and specimen from the Indian River after Bouin fixation (**G**). (A, B) Fully extended specimens. The distance between the elongated anteriormost cilia of the right and left ciliary fields (arrows) and the membranellar zone is extraordinarily large. Arrowhead (A) marks the lateral ciliary field. (C, G) Not fully contracted specimens. (D, F) Optical longitudinal section and surface view of a maximally contracted specimen in a lorica with a distinctly flared collar. (E) Detail of the peristomial rim showing the clavate tentaculoids. A, collar annuli; CM, collar membranelles; L, lorica; PE, peduncle; SC, somatic cilia; T, tentaculoids; VK, ventral kinety. Scale bars = 30 μm (A–D, F, G), 10 μm (E).

**Figure 3:**
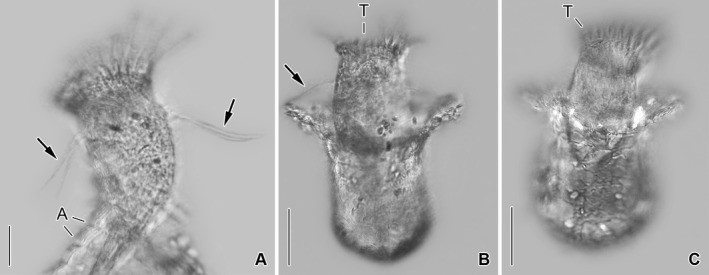
*Tintinnopsis everta*, specimens from the Indian River from life. Like in the specimens from Maryland, the elongated anteriormost cilia (arrows; **A**,** B**) of the right and left ciliary fields insert distinctly apart from the collar membranelles and club‐shaped tentaculoids are between the collar membranelles (B, **C**). A, collar annuli; T, tentaculoids. Scale bars = 10 μm (A), 30 μm (B, C).

**Figure 4:**
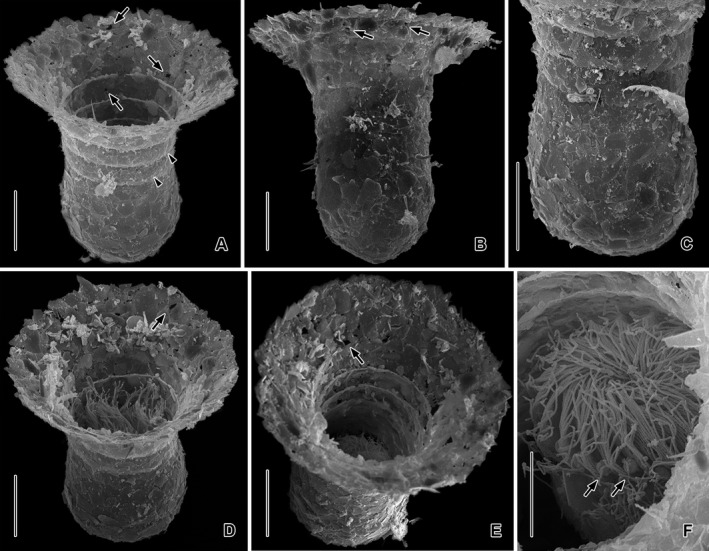
*Tintinnopsis everta*, specimens from the inlet in Ocean City in the scanning electron microscope. (**A–E**) Oblique top views (A, D, E), lateral view (B), and posterior portion (C) of loricae. Although the loricae are comparatively hyaline in the light microscope, the SEM micrographs show mineral particles agglutinated not only on the outer but also on the inner surface of the collar, which has minute holes (arrows; A, B, D, E). The collar shows slightly projecting rims on the inner surface, which correspond to shallow furrows on the outer surface (arrowheads; A). The lorica bowl is subspherical and has very thin mineral particles agglutinated (B, C). (**F**) Contracted specimen. Clavate tentaculoids insert in the outer portions of the intermembranellar ridges (arrows). Scale bars = 20 μm (A–E), 10 μm (F).

Lorica 61–115 μm long, agglutinated, campanulate, i.e. composed of a subspherical bowl and a funnel‐shaped collar, without posterior process; ratio of length to opening diameter 0.9–1.3:1 (Fig. 1A, 2A–G, 3A–C, 4A–E); slightly deformed in protargol slides. Bowl 40–64 μm long, occupying about 58% of lorica length, 40–75 μm wide in protargol slides (39–51 μm, $\overset{¯}{x}$ = 42 μm in SEM micrographs and in vivo; *n* = 9), often slightly wider than cylindroidal collar portion (narrowest lorica portion); posterior end broadly rounded, with an angle of 51–59° in SEM micrographs, rarely tapered. Collar rather variable in length (26–60 μm), composed of a flared anterior portion with an irregular opening rim 58–94 μm across (46–88 μm, $\overset{¯}{x}$ = 72 μm in SEM micrographs and in vivo; *n* = 10) and a cylindroidal posterior portion 44–63 μm wide in protargol slides (32–41 μm, $\overset{¯}{x}$ = 37 μm in SEM micrographs and in vivo; *n* = 8); angle between anterior end and flared collar portion about 58° in SEM micrographs. Three to five convex annuli in posterior collar portion, each 7–10 μm high, indistinct in protargol‐stained material, while clearly visible in live specimens and SEM micrographs (Fig. 1A, 2A, B, 3A, 4A–F). Lorica wall comparatively hyaline because matrix layer and agglutinated particles are thin (about 1 μm thick in SEM micrographs) and do not form a continuous wall in the flared collar portion; opening rim and seams between collar annuli more refractive due to more dense agglutination (see below). Inner wall of bowl with smooth lining, while that of collar rough owing to agglutination of particles and low horizontally orientated circular projections (Fig. 4A, D–F). Inner projections and outer furrows between collar annuli result from mode of collar formation: a slightly convex ring inserts somewhat subapically on the outer surface of the previously formed (posterior) ring; hence, rings overlap to a certain degree, rendering these lorica portions darker under the light microscope (Fig. 1A, 2A, B, 3A). Agglutinated particles of abiotic (mineral particles), rarely biotic (e.g. fragments of diatom frustules) origin, larger on bowl (up to 8 μm across) than on collar.

Cell proper of fully extended live specimen usually 50–60 × 25–30 μm in size and elongate obconical, gradually merges into slender, wrinkled, and highly contractile peduncle up to 60 × 3 μm in size attached to bottom of lorica; cell protrudes conspicuously far beyond opening rim (Fig. 1A, 2A, B, E, 3A–C). In disturbed or preserved specimens, cell proper contracted by about 50% and almost globular, measuring about 32 μm across (Fig. 1B–E, 2D, 6A–F). Invariably two macronucleus nodules, usually in posterior 75% of cell proper, 7–15 × 4–9 μm in size after protargol staining, usually broadly ellipsoidal to ovoidal, with nucleoli 0.5–1.5 μm across; anterior nodule often parallel to main cell axis, while posterior nodule frequently horizontally orientated, both generally connected by thin isthmus (Fig. 1C, G). Two, rarely one or three micronuclei adjacent to macronucleus nodules, about 1 μm across, faintly impregnated with protargol. Contractile vacuole, cytopyge, striae, and accessory combs neither recognised in live nor in preserved material. Tentaculoids originate in outer portions of intermembranellar ridges, recognisable in vivo (found in three out of four specimens), while possibly contracted or too hyaline to be visible in protargol‐stained material, clavate to pin‐shaped, about 3 × 1.5 μm in size (Fig. 1A, 2E, 3B, C, 4F). Capsules and myonemes not stained with protargol. Cytoplasm colourless and granular, contains food vacuoles up to 5 μm across with green flagellates or pennate diatoms. Living cell shows pumping movement of peristomial field and swims by rotation about main cell axis (speed not measured), twitches back on obstacles. Disturbed individuals retract into lorica with motionless membranelles bent to centre of peristomial field (Fig. 2C, D, 4E, F, 6A–F).

**Figure 5:**
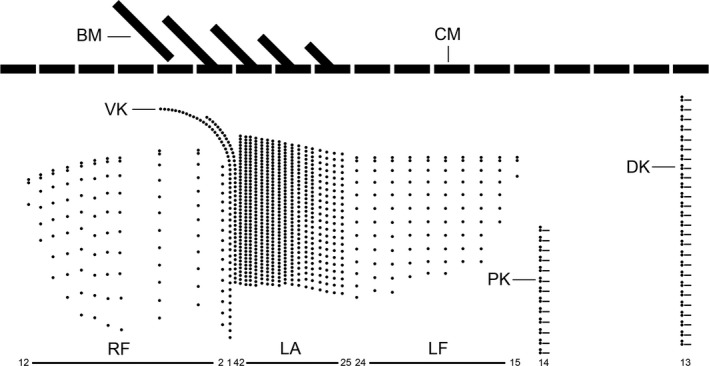
*Tintinnopsis everta*, kinetal map based on data from morphostatic specimens from the Chesapeake Bay. Cilia are shown only in the dorsal and posterior kineties; otherwise, all basal bodies are ciliated. Note the conspicuously large distance between the ciliary fields and the membranellar zone as well as the unique position of the posterior kinety. BM, buccal membranelle; CM, collar membranelles; DK, dorsal kinety; LA, lateral ciliary field; LF, left ciliary field; PK, posterior kinety; RF, right ciliary field; VK, ventral kinety.

**Figure 6:**
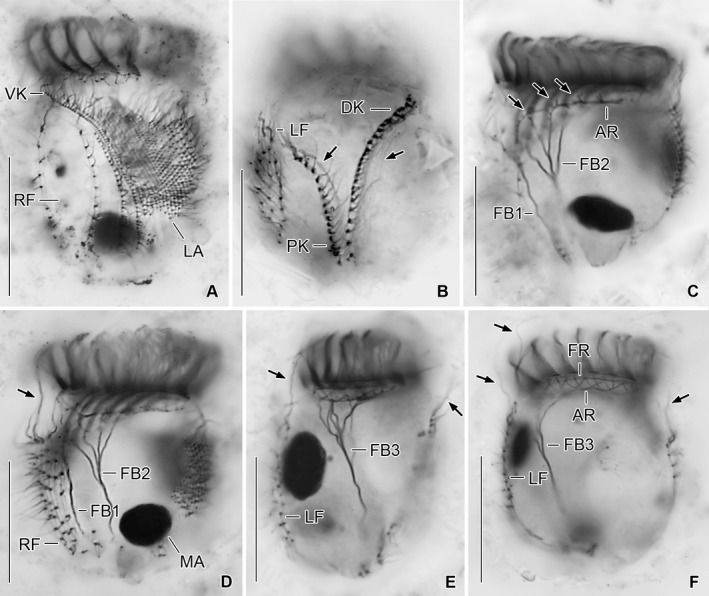
*Tintinnopsis everta*, specimens from the Chesapeake Bay after protargol staining. (**A**) Ventral view showing the extraordinarily wide spacing of the first rows in the right field. (**B**) Dorsal view showing the unique position of the posterior kinety. The arrows indicate fibre bundles accompanying the dorsal and posterior kineties. (**C**,** D**) Optical longitudinal sections showing the buccal cavity and the fibre bundles originating in the buccal membranelle and the elongated collar membranelles; possibly, the fibre bundles are also connected with the adoral ring (arrows; C). The anteriormost cilia of the right field are elongated (arrow; D). (**E**,** F**) Optical longitudinal sections. Fibre bundles originate in the dorsal portion of the adoral ring. Arrows mark the elongated anteriormost cilia in the right and left ciliary fields. A complex system of argyrophilic structures/fibres is associated with the adoral zone of membranelles (F). AR, adoral ring; DK, dorsal kinety; FB1, fibre bundle originating in buccal membranelle; FB2, fibre bundle originating in elongated collar membranelles; FB3, fibre bundle originating in dorsal portion of adoral ring; FR, fibrillar ring; LA, lateral ciliary field; LF, left ciliary field; MA, macronucleus nodule; PK, posterior kinety; RF, right ciliary field; VK, ventral kinety. Scale bars = 20 μm.

Somatic ciliary pattern of most complex type (Agatha and Strüder‐Kypke 2007), i.e. it comprises a ventral, dorsal, and posterior kinety as well as a right, left, and lateral ciliary field (Table 1 and Fig. 1B, C, 5, 6A, B). Kineties usually highly variable in lengths probably because of basal body proliferation or resorption in late dividers and/or postdividers; generally, comparatively long, especially ventral kinety, terminating in posterior half or third of cell proper. Kinetids of each ciliary row ostensibly connected by an argyrophilic fibre (probably postciliary microtubules). Ventral kinety commences about 4 μm posteriorly to collar membranelles and anteriorly to third, occasionally fourth kinety of right ciliary field, curves leftwards and extends parallel to kineties of lateral ciliary field to posterior end of cell proper, composed of monokinetids densely spaced in anterior, but more widely spaced in posterior portion (Fig. 1A, B, 5, 6A); kinetid numbers not estimated because of dense spacing. Cilia of ventral kinety 4–5 μm, rarely 7–8 μm long after protargol staining. Right ciliary field separated from collar membranelles by conspicuously broad unciliated stripe increasing in width from about 9 μm at the left end to about 12 μm at the right end in stained specimens, comprises 9–12 kineties (Fig. 1A, B, 5, 6A, D). First four kineties extremely widely spaced, i.e. distance between first and second row about 3 μm and between second, third, and fourth row about 5 μm each. Last four kineties gradually decrease in length in clockwise direction (top view). Kineties composed of monokinetids and one anterior dikinetid, except for (i) first row exclusively composed of densely spaced monokinetids starting 1–2 μm posteriorly to second kinety and (ii) second kinety with rarely two anterior dikinetids (two out of 18 specimens). Cilia of right field about 5 μm long in vivo and 4–6 μm in protargol‐stained cells, except for elongated anterior cilia of dikinetids (soies; Fig. 1A, 2A, B, 3A, B, 6D–F; Fauré‐Fremiet 1924) measuring about 28 μm in vivo and about 21 μm after protargol staining. Dorsal kinety commences about 3 μm posteriorly to collar membranelles and thus more anteriorly than ciliary fields; extends in leftward curvature to posterior end of cell proper, composed of 19–34 dikinetids having a cilium about 7 μm long (after protargol staining) associated only with each posterior basal body (Fig. 1C, 5, 6B, 7C). Posterior kinety commences about 18 μm posteriorly to collar membranelles, about 16 μm apart from dorsal kinety, and about 2 μm apart from left ciliary field, performs rightward curvature, and extends with posterior portion parallel to dorsal kinety, terminating near posterior end of cell proper, composed of 12–17 dikinetids having a cilium about 7 μm long (after protargol staining) associated only with each posterior basal body (Fig. 1C, 5, 6B, 7C). Four argyrophilic structures of probably fibrillar nature extend parallel to the right sides of dorsal and posterior kineties (Fig. 6B, 7C). Left ciliary field separated from collar membranelles by conspicuously broad (about 10 μm) unciliated stripe, composed of ten or eleven kineties that gradually increase in length in clockwise direction (top view), composed of monokinetids and one anterior dikinetid (Fig. 1A–C, 5, 6B, E, F). Cilia of left field about 5 μm long in vivo, while 4–6 μm after protargol staining, except for elongated anterior cilia of dikinetids (soies; Fig. 1A, 2A, B, 3A, B, 6E, F; Fauré‐Fremiet 1924) measuring about 28 μm in vivo and about 21 μm after protargol staining. Lateral ciliary field separated from collar membranelles by conspicuously broad unciliated stripe decreasing in width from about 9 μm at the left end to about 7 μm at the right end, except for last kinety which commences about 5 μm posteriorly to collar membranelles and anteriorly to second kinety of right field, extending at a distance of about 0.5 μm anteriorly and parallel to the distinctly curved ventral kinety (Fig. 1A, B, 5, 6A). Kineties monokinetidal, more densely spaced in right than in left field portion. Cilia of lateral field about 5 μm long in right kineties, while conspicuously long (about 10 μm after protargol staining) in anterior portion of left kineties (Fig. 1A).

**Table 1:** Morphometric data of *Tintinnopsis everta* from the Chesapeake Bay (ML, USA), except for the annuli numbers which are from SEM micrographs and Bouin‐fixed material collected in Ocean City

Characteristics^a^	$\overset{¯}{x}$	M	SD	SE	CV	Min	Max	*n*
Lorica, total length	81.5	77.5	14.3	2.5	17.6	61.0	115.0	32
Lorica, bowl length	51.4	50.0	6.1	1.6	11.8	40.0	64.0	15
Lorica, bowl width^d^	55.3	52.0	10.3	2.1	18.6	40.0	75.0	23
Lorica total length:bowl length, ratio	1.7	1.6	0.2	0.1	13.5	1.4	2.2	14
Lorica total length:bowl length, per cent	57.8	58.3	6.9	1.8	12.0	45.5	67.9	14
Lorica, collar length	38.6	33.0	12.4	3.2	32.0	26.0	60.0	15
Lorica opening diameter, width^d^	71.5	67.0	10.5	1.9	14.7	58.0	94.0	31
Lorica diameter of cylindroidal portion^b^^,^ d	54.7	55.0	5.6	1.2	10.2	44.0	63.0	21
Lorica total length:opening diameter, ratio^d^	1.1	1.1	0.1	0.0	8.7	0.9	1.3	31
Lorica, number of annulations (from SEM and Bouin)	4.1	4.0	0.6	0.2	14.6	3.0	5.0	9
Cell proper, length	32.4	33.0	3.0	0.5	9.4	26.0	40.0	32
Cell proper, width	32.0	32.0	3.9	0.7	12.2	23.0	44.0	32
Cell proper length:width, ratio	1.0	1.0	0.1	0.0	12.1	0.8	1.3	32
Macronucleus nodules, number	2.0	2.0	0.0	0.0	0.0	2.0	2.0	30
Anterior macronucleus nodule, length	10.1	10.0	1.6	0.3	15.9	7.0	15.0	30
Anterior macronucleus nodule, width	6.3	6.0	1.1	0.2	18.2	4.0	9.0	30
Anterior cell end to anterior macronucleus nodule, distance	8.1	8.0	2.2	0.4	27.3	5.0	13.0	28
Micronuclei, number	2.0	2.0	0.4	0.1	18.3	1.0	3.0	16
Micronucleus, diameter	1.0	1.0	0.0	0.0	0.0	1.0	1.0	16
Ventral kinety, length	29.9	30.0	3.6	1.1	12.1	25.0	35.0	10
Ventral kinety, distance to collar membranelles	4.4	4.0	1.9	0.4	44.1	2.0	9.0	24
Dorsal kinety, length^c^	27.9	28.0	2.2	0.5	8.0	24.0	33.0	21
Dorsal kinety, number of dikinetids	27.0	27.0	4.2	0.9	15.6	19.0	34.0	21
Dorsal kinety, distance to right ciliary field^e^	5.6	5.0	2.1	0.5	38.1	3.0	12.0	22
Dorsal kinety, distance to collar membranelles	3.0	3.0	1.4	0.2	44.8	1.0	7.0	33
Posterior kinety, length^c^	14.9	15.0	2.2	0.5	14.9	12.0	20.0	21
Posterior kinety, number of dikinetids	14.5	15.0	1.5	0.3	10.2	12.0	17.0	21
Posterior kinety, distance to dorsal kinety^e^	16.0	16.0	3.2	0.7	20.1	10.0	22.0	22
Posterior kinety, distance to left ciliary field^e^	2.6	2.0	1.5	0.3	56.4	1.0	6.0	20
Posterior kinety, distance to collar membranelles	18.2	17.5	4.0	0.7	21.8	11.0	26.0	30
Right ciliary field, number of kineties	10.7	11.0	0.8	0.2	7.7	9.0	12.0	14
Kinety 1 in right ciliary field, length	18.9	19.0	3.3	0.9	17.2	13.0	23.0	12
Kinety 1 in right ciliary field, number of kinetids	20.3	20.0	2.8	0.9	14.0	14.0	24.0	11
Kinety 1 in right ciliary field, number of dikinetids	0.0	0.0	0.0	0.0	0.0	0.0	0.0	17
Kinety 1 in right ciliary field, distance to collar membranelles	10.2	10.0	2.8	0.5	27.5	6.0	17.0	26
Kinety 2 in right ciliary field, length	17.9	19.0	2.1	0.6	11.5	15.0	21.0	13
Kinety 2 in right ciliary field, number of kinetids	11.8	12.0	2.3	0.6	19.7	7.0	14.0	13
Kinety 2 in right ciliary field, number of dikinetids	1.1	1.0	0.3	0.1	29.1	1.0	2.0	18
Kinety 2 in right ciliary field, distance to collar membranelles	8.6	9.0	2.4	0.5	28.2	5.0	16.0	23
Kinety 4 in right field, length	16.5	16.0	1.1	0.3	6.5	15.0	19.0	10
Kinety 4 in right field, number of kinetids	8.4	8.0	2.1	0.7	25.2	6.0	13.0	10
Kinety 4 in right field, distance to collar membranelles	9.5	9.0	2.0	0.4	21.3	5.0	14.0	29
Kinety *n* in right field, length	3.3	3.0	0.8	0.2	23.2	2.0	5.0	12
Kinety *n* in right field, number of kinetids	1.6	1.0	0.8	0.2	50.1	1.0	3.0	12
Kinety *n* in right field, distance to collar membranelles	12.1	12.0	1.6	0.3	13.0	10.0	15.0	27
Lateral ciliary field, number of kineties	17.5	17.5	1.0	0.3	5.7	16.0	19.0	12
Lateral ciliary field, width	12.0	12.0	1.3	0.4	11.1	9.0	14.0	10
Kinety 1 in lateral field, length	15.8	16.0	1.1	0.4	7.2	14.0	17.0	10
Kinety 1 in lateral field, distance to collar membranelles	9.1	9.0	2.9	0.5	32.3	4.0	18.0	29
Kinety *n* − 1 in lateral field, length	15.0	15.0	1.6	0.5	10.9	11.0	17.0	10
Kinety *n* − 1 in lateral field, distance to collar membranelles	7.0	7.0	2.4	0.5	34.4	4.0	14.0	25
Kinety *n* in lateral field, length	19.0	19.0	1.7	0.6	8.9	16.0	21.0	8
Kinety *n* in lateral field, distance to collar membranelles	5.1	5.0	2.2	0.5	41.8	3.0	12.0	21
Left ciliary field, number of kineties	10.2	10.0	0.4	0.1	4.3	10.0	11.0	13
Kinety 1 in left field, length^f^	2.9	3.0	0.9	0.3	32.4	2.0	5.0	11
Kinety 1 in left field, number of kinetids	1.8	2.0	0.6	0.2	33.2	1.0	3.0	11
Kinety 1 in left field, distance to collar membranelles	9.5	10.0	2.3	0.5	24.5	5.0	13.0	21
Kinety *n* in left field, length	16.2	16.0	2.1	0.7	12.9	13.0	19.0	10
Kinety *n* in left field, number of kinetids	14.2	15.5	3.3	1.0	23.0	8.0	19.0	10
Kinety *n* in left field, distance to collar membranelles	9.5	9.5	2.9	0.6	30.8	4.0	17.0	22
Adoral zone of membranelles, diameter	26.0	26.0	1.6	0.4	6.3	23.0	28.0	15
Collar membranelles, number	18.5	19.0	0.5	0.2	2.8	18.0	19.0	11
Collar membranelles, number of elongated ones	4.0	4.0	0.0	0.0	0.0	4.0	4.0	10
Buccal membranelle, number	1.0	1.0	0.0	0.0	0.0	1.0	1.0	10
Cilium in anterior left portion of lateral field, length	9.8	10.0	0.7	0.2	6.8	8.0	10.0	9
Cilium in right portion of lateral field, length	4.9	5.0	1.0	0.2	20.4	4.0	7.0	10
Cilium of monokinetids in right and left fields, length	5.0	5.0	0.5	0.1	10.7	4.0	6.0	25
Cilium of dikinetids in right and left fields, length	20.7	20.0	3.1	0.6	15.0	14.0	26.0	27
Cilium in posterior kinety, length	7.0	7.0	1.0	0.2	13.9	6.0	8.0	18
Cilium in dorsal kinety, length	6.9	7.0	0.9	0.2	13.5	6.0	8.0	18

a Data based—if not stated otherwise—on protargol‐stained, mounted, and randomly selected specimens from field material. Measurements in μm. CV, coefficient of variation in %; M, median; Max, maximum; Min, minimum; *n*, number of individuals investigated; SD, standard deviation; SE, standard error of arithmetic mean; $\overset{¯}{x}$, arithmetic mean.

b Width of posteriormost annulus.

c Measured as cord of organelle.

d Lorica slightly deformed in protargol preparations and relatively indistinct; width measurements should thus be used with caution. Preferably, use SEM and life data from text.

e Distance between anterior end of kinety and particular structure.

f Length only measured when kinety comprised two or more kinetids.

**Figure 7:**
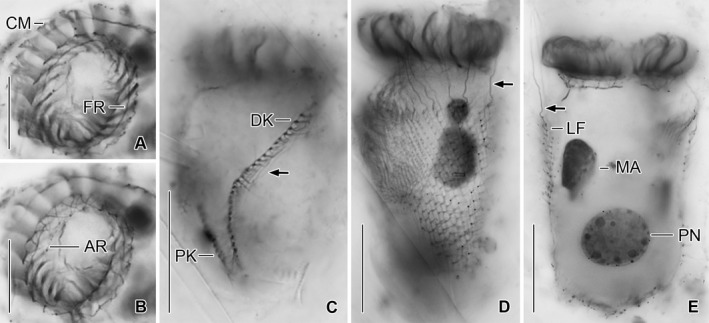
*Tintinnopsis everta*, specimens from the Chesapeake Bay after protargol staining. (**A**,** B**) Top views of same specimen at different focal planes. For explanation of the fibrillar associates of the membranellar zone see text and Fig. 1H. (**C**) Four parallel argyrophilic fibres (arrow) are apparently associated with the dorsal kinety. (**D**,** E**) Specimens infected by an unknown endoparasite at different focal planes. Arrows mark the elongated anteriormost cilia of the right and left ciliary fields. AR, adoral ring; CM, collar membranelles; DK, dorsal kinety; FR, fibrillar ring; LF, left ciliary field; MA, macronucleus nodule; PK, posterior kinety; PN, parasite's nucleus. Scale bars = 15 μm (A, B), 20 μm (C–E).

Oral apparatus occupies anterior cell portion. Adoral zone of membranelles closed, 23–28 μm across in vivo and after protargol staining, perpendicular to main cell axis in contracted specimens, composed of 18 or 19 collar membranelles and invariably one buccal membranelle (Fig. 1A–E, I, 2A–D, G, 3A–C, 4F, D, 5, 6A–F). Collar membranelles up to 26–34 μm long in vivo, triangular, i.e. cilia decrease in length from outer to inner end of membranelles. Distal membranellar portions frayed, producing a comb‐like appearance (Fig. 1A). Polykinetids of collar membranelles extend obliquely across peristomial rim, forming a contorted pattern (Stearn 2004), separated by shallow ridges, comprise three rows of basal bodies. Polykinetids of proximalmost four collar membranelles successively elongated, terminating 4–8 μm posteriorly to apical cell end in buccal cavity along with buccal membranelle (Fig. 1D, E, I, 5, 6C, D). Complex system of argyrophilic structures/fibres associated with adoral zone of membranelles, comprises four circular structures and some bundles originating in the collar polykinetids (Fig. 1H, I, 6F, 7A, B); transmission electron microscopic data are needed for verification. Two circular fibres connect inner and outer ends of collar polykinetids (Fig. 1H, black fibres). Four argyrophilic fibre bundles originate from each collar polykinetid, optically cross bundles of adjacent membranelles, and merge into a horizontally orientated circular fibre underneath the membranellar zone, the so‐called adoral ring (Fig. 1D, E, H, I, 6C, F, 7A, B; Campbell 1926): two long bundles originate at the outer end of each polykinetid and extend in clockwise (Fig. 1H, green fibre) and counter‐clockwise (Fig. 1H, red fibre) direction, terminating near inner end of same polykinetid and second previous polykinetid, respectively; third bundle long, originates in the polykinetid's middle portion and extends counter‐clockwise (Fig. 1H, blue fibre), terminating near inner end of second previous polykinetid; and fourth bundle short, commences at the polykinetid's inner end and extends in slightly clockwise direction (Fig. 1H, yellow fibre). Fourth ring‐shaped argyrophilic structure, the fibrillar ring, extends in centre of peristomial rim (Fig. 1E, H, I, 6F, 7A) and is apparently not associated with any other of the previously mentioned structures. Further fibre bundles originate from proximal portions of elongated collar polykinetids and buccal polykinetid and extend longitudinally posteriorly, terminating near end of cell proper; those from the elongated collar polykinetids fuse to one bundle (FB2; Fig. 1D, E, 6C, D), while that of the buccal polykinetid remains separate (FB1); possibly, the bundles are also connected with the adoral ring (Fig. 6C). Similar fibre bundles originate in dorsal portion of the adoral ring adjacent to the distal end of the endoral membrane and fuse to one bundle extending to posterior end of cell proper (FB3; Fig. 1C, E, H, 6E, F). Endoral membrane commences in dorsal portion of peristomial field and extends in short distance parallel to membranellar zone into buccal cavity; composed of a single row of basal bodies, probably with monostichomonad structure (Fig. 1C, E, H, I). Pharyngeal fibres about 5 μm long in protargol‐stained specimens, extend obliquely posteriorly (Fig. 1D); their origin is uncertain.

### Ontogenesis

About 20 early, five middle, and two late dividers were found. *Tintinnopsis everta* shows an enantiotropic division mode with hypoapokinetal stomatogenesis in a subsurface pouch in the posterior half of cell proper, i.e. left of ventral kinety and posteriorly to the last lateral rows. The somatic kineties elongate by intrakinetal proliferation.

**Figure 8:**
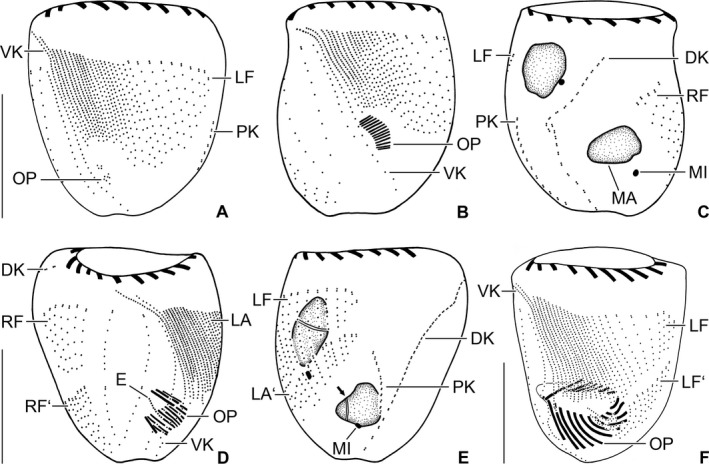
*Tintinnopsis everta*, dividers from the Chesapeake Bay after protargol staining. (**A**) Ventral view of a very early divider. (**B**,** C**) Ventral and dorsal views of same early divider. The split of the dorsal kinety is probably an artefact because no other kinety shows signs of division or even distinct basal body proliferation. (**D**,** E**) Ventral and dorsal views of same early middle divider showing replication bands (arrow; E). (**F**) Ventral view of a middle divider. E, opisthe's endoral membrane; DK, dorsal kinety; LA, LA’, proter's and opisthe's lateral ciliary fields; LF, LF’, proter's and opisthe's left ciliary fields; MA, macronucleus nodules; MI, micronuclei; OP, oral primordium; PK, posterior kinety; RF, RF’, proter's and opisthe's right ciliary fields; VK, ventral kinety. Scale bars = 20 μm.

**Figure 9:**
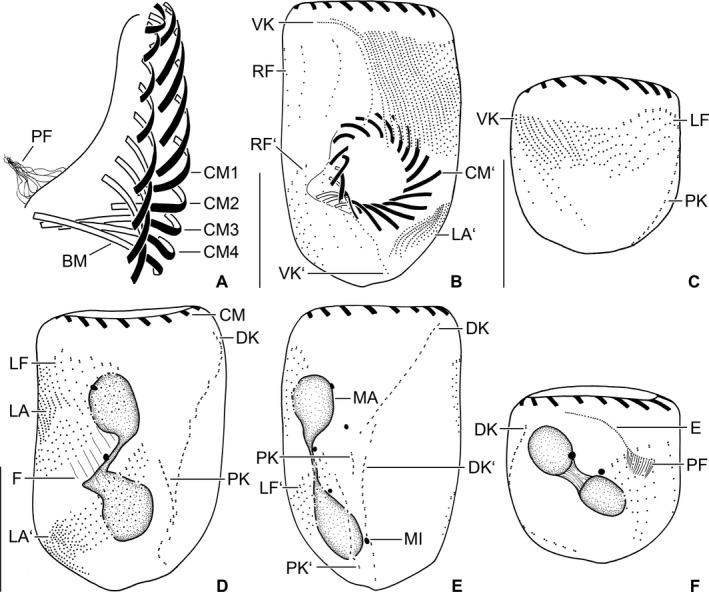
*Tintinnopsis everta*, dividers from the Chesapeake Bay after protargol staining. (**A**) Right lateral view of the opisthe's oral apparatus in a late middle divider. (**B**,** E**) Ventral and dorsal views of same late middle divider. Interestingly, five instead of the four expected division products of the micronuclei are visible. (**C**,** F**) Ventrolateral and dorsolateral views of same opisthe postdivider. (**D**) Dorsolateral view of a late middle divider. Apparently, the micronuclei have not split as yet. BM, buccal membranelle; CM, CM’, proter's and opisthe's collar membranelles; CM1‐4, elongated collar membranelles 1–4 extending into the buccal cavity; DK, DK’, proter's and opisthe's dorsal kineties; E, endoral membrane; F, argyrophilic fibres; LA, LA’, proter's and opisthe's lateral ciliary fields; LF, LF’, proter's and opisthe's left ciliary fields; MA, macronucleus nodules; MI, micronuclei; PF, probably future pharyngeal fibres; PK, PK’, proter's and opisthe's posterior kineties; RF, RF’, proter's and opisthe's right ciliary fields; VK, VK’, proter's and opisthe's ventral kineties. Scale bars = 20 μm.

The adoral membranelles immediately commence to differentiate in the cuneate field of anarchic basal bodies in early dividers (Fig. 8A–C). The new funnel‐shaped membranellar zone is perpendicularly orientated to the cells ventral side in middle dividers (Fig. 8D, F). Finally, the opisthe's dorsal side faces the proter's ventral side (Fig. 9A, B).

Basal body proliferation and division of kineties first occur in the right and left ciliary fields, while these processes take place somewhat later in the lateral field (Fig. 8D–F). In late middle dividers, argyrophilic structures/fibres still seem to connect the corresponding kinety fragments of proter and opisthe (Fig. 9D). The opisthe's ventrally located rows arrange in a semi‐circle around the lower margin of the developing oral primordium (Fig. 9B). Since only a single late middle divider displaying the splits of dorsal and posterior kineties could be studied, it cannot be excluded that it shows a non‐representative pattern (Fig. 9B, E) as five kinety fragments are recognisable (listed from right to left): (i) a short fragment with three dikinetids separated by the future division furrow from the opisthe's dorsal kinety; (ii) the long opisthe's dorsal kinety; (iii) the long proter's dorsal kinety; (iv) a short fragment with four dikinetids which is separated by the future division furrow from the proter's dorsal kinety; and (v) the long opisthe's posterior kinety. While the long fragments are reliably identified, the occurrence of fragment (i) and the position of fragment (iv), which should represent the short proter's posterior kinety, are peculiar; their affiliation/origin cannot be elucidated due to the too faint impregnation or absence of fibres connecting the kinetids. Actually, the uncommon position of the latter fragment might be a preparation artefact. In contrast to the posterior kinety and probably the dorsal kinety, the divisions of all other ciliary rows produce larger fragments for the proter than for the opisthe (Fig. 9B, D, E).

One replication band each traverses the macronucleus nodules in early middle dividers (Fig. 8E) that afterwards fuse to a longitudinally orientated elongate ellipsoidal mass. In late middle dividers, the mass and the micronuclei split (Fig. 9D, E). Simultaneously, the dorsal and ventral fibre bundles commence to disintegrate at their proximal ends, and the cortex widens in longitudinal direction between the proter's and opisthe's kineties, forming a broad blank stripe for the future division furrow, especially on ventral side (Fig. 9B, D).

One early postdivider, namely, an opisthe in a lorica, was available (Fig. 9C, F). It demonstrates that morphogenesis and reconstruction of the interphase nuclear apparatus is not finished (at least in this division product) with the separation of proter and opisthe. Its ventral kinety and last lateral kinety are almost straight, and the former commences only anteriorly to the first row of the right ciliary field; accordingly, the conspicuous anterior elongation of these two rows takes place only in late opisthe postdividers. All ciliary fields are still shorter than in morphostatic specimens, indicating the need of a second round of intrakinetal proliferation in late opisthe postdividers. The dividing macronucleus nodule suggests that the specimen has just separated from the proter. The dorsal and ventral fibre bundles have not formed as yet. Lorica formation has not been observed.

### Molecular characteristics and phylogenetic placement

The partial SSU rRNA gene sequence is 1,390 nucleotides long with a GC content of 48.2% and has been deposited in GenBank under the accession number MG461220. The distance data reveal 7.7–8.6% divergence to the *Tintinnopsis* species of tintinnid clade 1 and 4–6.5% divergence to *Tintinnopsis* species of tintinnid clades 2–11.

**Figure 10:**
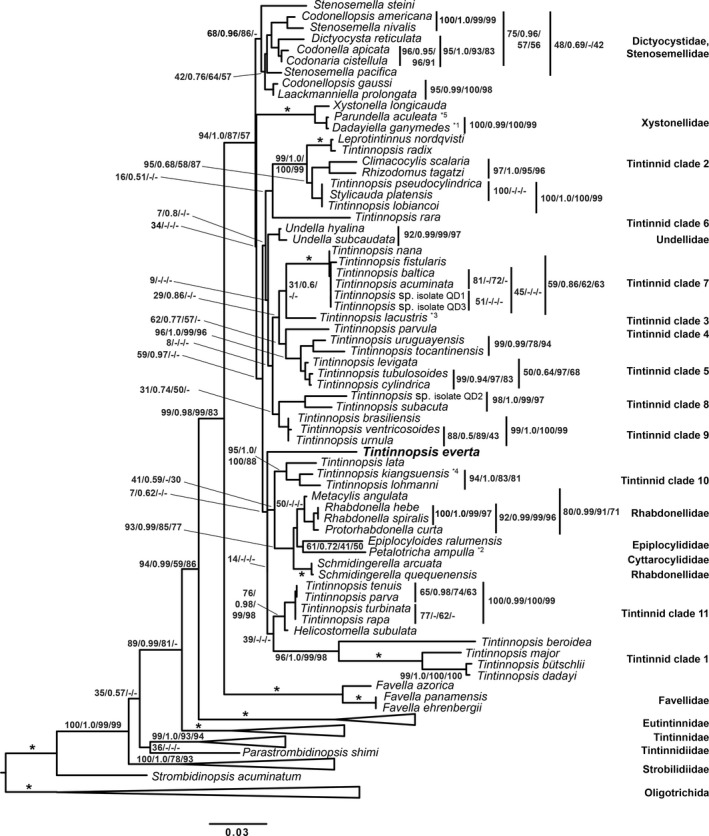
Small subunit (SSU) rRNA phylogenetic reconstruction of tintinnid phylogeny computed with RAxML based on the GTR + I + Γ model. The first number at the nodes represents the bootstrap support for RAxML (ML); the second number represents posterior probability values of the Bayesian Inference analysis (BI); and the third and fourth numbers represent bootstrap values for Neighbor Joining (NJ) and Maximum Parsimony (MP), respectively. Asterisks indicate full support in all analyses; dashes indicate support values below 25% and 0.5 posterior probability (although all low values for the ML analysis are shown). The scale bar represents 3 substitutions per 100 nucleotides. Numbering of tintinnid clades follows Santoferrara et al. (2017). The GenBank accession numbers are listed in the supplementary Table S1. *^1^ *Dadayiella ganymedes* had probably been confused with *D. bulbosa*; *^2^ should possibly be affiliated with genus *Cyttarocylis* (Dolan et al. 2014); *^3^according to Foissner et al. (1999) a synonym of *Codonella cratera*; *^4^possibly the senior synonym of *Stenosemella lacustris*; *^5^ the sequenced specimen was misidentified as suggested by Agatha and Strüder‐Kypke (2014) and confirmed by Santoferrara et al. (2017), it is probably conspecific with *Dadayiella acutiformis* Kofoid and Campbell, 1939.

The four phylogenetic analyses resulted in trees differing in the placement of *T. everta* as well as in the topology of most tintinnid clades. The ML tree grouped *T. everta* together with the tintinnid clades 1, 10, 11, the genera *Epiplocyloides* and *Petalotricha*, and the family Rhabdonellidae (Fig. 10). In the BI and MP analyses, *T. everta* is an adelphotaxon to tintinnid clade 1 (Fig. S1, S2), while the NJ analysis placed *T. everta* as adelphotaxon to the tintinnid clades 3–11, the Undellidae, and the families aforementioned (Fig. S3). However, none of the nodes at this level showed any significant support (Fig. 10). Depending on outgroup composition (i.e. exclusion of long‐branch species like *Parastrombidinopsis shimi*,* Strombidinopsis acuminata*, and *Novistrombidium testaceum*), trees were more congruent and robust (data not shown), placing *T. everta* into a group with tintinnid clades 10, 11, the Rhabdonellidae, *Epiplocyloides*, and *Petalotricha*. Since support values in those trees were also low, and for better comparison with published phylogenies, we decided to rather provide the contradicting trees instead. Thus, the phylogenetic placement of *T. everta* cannot be resolved with the data currently available.

### Infection

Four specimens from the Chesapeake Bay, including an early divider, were found to be infected by an unknown parasite of which only the nucleus was visible. The supposed parasite is in the posterior third of cell proper and surrounded by a lightly stained area about 1 μm wide. The almost globular nucleus is 8–15 μm across and contains nucleoli with a diameter of up to 2 μm. The infection apparently causes several changes in the host: (i) a longitudinal orientation of the posterior macronucleus nodule; (ii) a deformation of the nodules (Fig. 7D, E); (iii) an increase in cell length (up to twice the common length); and (iv) an elongation of the somatic kineties by basal body proliferation. The parasite's nucleus occupies up to 30% of the host cell in presumed late infection stages.

## Discussion

### Comparison with original description

The specimens collected at the three sampling sites match in lorica features, especially the flared collar with annuli, and the uniquely large distance between the membranellar zone and the ciliary fields; hence, conspecificity of the populations is beyond question.

*Tintinnopsis baltica* var. *rotundata* was discovered by Laackmann (1908) and raised to rank of a species with the introduction of the replacement name *Tintinnopsis everta* by Kofoid and Campbell (1929), avoiding homonymy with *Tintinnopsis rotundata* Jörgensen, 1899. The original description is based on specimens from the Baltic Sea and focuses on lorica characteristics, but also mentions the nuclear apparatus to be composed of two macronucleus nodules and two micronuclei (Fig. 1F; Laackmann 1908). The posteriorly rounded bowl merges into a cylindroidal collar portion, which more or less gradually widens forming a flared apical collar portion that clearly exceeds the bowl in diameter; a posterior process is absent. The lorica is 65–81 μm long and has an opening diameter of 50–52 μm. The cylindroidal collar portion is about 35 μm across, the bowl width is about 32 μm, and the collar length is about 28 μm; these data have been inferred from the single illustration like the four annuli in the collar and the angle of the broadly rounded bowl (about 60°).

The features of the Baltic Sea specimens described by Laackmann (1908) match those of the specimens studied here very well (lorica length: 61–115 μm; bowl width: 39–75 μm; opening diameter: 46–94 μm; diameter of cylindroidal portion: 32–63 μm; 3–5 collar annuli; height of the collar annuli about 7 μm). Using Laackmann's illustration, Kofoid and Campbell (1929) also estimated the angle between the anterior end and the flared collar, which is very similar to that measured in our SEM micrographs (55° vs. about 58° in specimens with rather well developed collars). Admittedly, the opening diameters are on average larger in our specimens than in those found in the Baltic Sea. However, the opening diameter is distinctly influenced by the length of the flared collar portion and slight differences in its angle; hence, we attribute the deviating mean values to the potentially smaller number of specimens studied by Laackmann. Accordingly, conspecificity with *Tintinnopsis everta* Kofoid and Campbell, 1929 is assumed. No further populations of the species have been described.

### Comparison with similar species

Circumscriptions of *Tintinnopsis* species are hampered by the scarce knowledge about cell features as well as the phenotypic plasticity of the loricae. So, the following comparisons can merely be based on lorica features, focussing on species that are similar to *T. everta* in an overall shape of the lorica and, especially the flared collar; data not provided by the authors were inferred from the information given. Since the opening diameter is highly variable in species with flared collars, data on the more reliable narrowest collar portions, which correspond rather well with the diameters of the adoral zone of membranelles, are also compared. Actually, there are eleven similar species (Fig. S7), for which we only mention the distinguishing features: *Tintinnopsis bacoorensis* (without annuli; Roxas 1941); *T. bütschlii* (lorica length 135–140 μm; ratio of lorica length to opening diameter about 1.3:1; 6–8 annuli; von Daday 1887; supposed synonym of *T. campanula*, see below); *T. compressa* (lorica opening smaller than or of same width as bowl; without flared collar and annuli; von Daday 1887); *T. dadayi* (without annuli; additional collars insert inside; Kofoid 1905); *T. directa* (ratio of lorica length to opening diameter 1.6–2.2:1; without annuli, but six spiral turns; Hada 1932); *T. major* (lorica length about 125 μm; ratio of lorica length to opening diameter about 2:1; 8 or 9 annuli; Meunier 1910); *T. manilensis* (without annuli; cylindroidal collar portion elongated and only 27 μm across; Roxas 1941); *T. mortensenii* (lorica length 41 μm; without annuli; Schmidt 1902); *T. orientalis* (flared collar slightly convex; without annuli; Kofoid and Campbell 1929); *T. patula* (without annuli; distinctly narrowed cylindroidal collar portion about 24 μm wide; Meunier 1910); *T. schotti* (lorica length 100–110 μm; without annuli; maximum bowl width in anterior lorica half; without cylindroidal collar portion; Brandt 1906, 1907). At the current state of knowledge, i.e. merely based on lorica features, it is impossible to decide whether the observed differences have to be regarded as distinguishing features or should be attributed to the intraspecific variability. Hence, further studies on the cell morphologies and barcoding are required for reliable species separations.

A complex ciliary pattern is not only found in *Tintinnopsis everta*, but also in several congeners like *T. fimbriata* (Agatha 2008), *T. parvula* (Agatha 2010), and *T. radix* (Jiang et al. 2012), as well as in the genera *Codonella* (Foissner and Wilbert 1979), *Codonellopsis* (Petz et al. 1995), *Cymatocylis* (Petz et al. 1995), *Rhizodomus* (Saccà et al. 2012), and *Stenosemella* (Agatha and Tsai 2008). However, the pattern of *T. everta* is unique, deviating from the previously known patterns in important details: (i) the position of the posterior kinety (commences right of the left ciliary field vs. posteriorly to the left or lateral ciliary fields), (ii) the structure of the first kinety in the right ciliary field (monokinetidal vs. monokinetidal plus 1–3 anterior dikinetids; verification by TEM is required in *T. everta*), (iii) the length of the ventral kinety (terminates near posterior end vs. the middle of cell proper), (iv) the distance between the first four kineties of the right field (enlarged vs. all kineties are equally distant), and (v) the distance between the collar membranelles and the ciliary fields (conspicuously large in vivo and protargol stains with a width of 7–14 μm vs. of only up to 4 μm, but usually not measured because too inconspicuous).

The nonmonophyly of the genus *Tintinnopsis* is not only revealed by molecular data currently displaying 11 clades in the small subunit rRNA gene tree (Bachy et al. 2012; Santoferrara et al. 2017; Snoeyenbos‐West et al. 2002), but also by cladistic analyses, in which at least four branches are characterised by distinct somatic ciliary patterns (Agatha and Strüder‐Kypke 2007, 2013, 2014). Since the pattern of the type species *Tintinnopsis beroidea* is unknown and the identification of the sequenced specimen is not reliable, a reasonable split of the genus is, however, currently impossible (Agatha and Strüder‐Kypke 2013). Nevertheless, the preliminary data suggest some diagnostic features for a future split of the genus *Tintinnopsis* (Agatha and Strüder‐Kypke 2007), and the monotypic genera *Rhizodomus* and *Stylicauda* are maintained to give home to genetically and morphologically distinct clades currently assigned to the genus *Tintinnopsis* (Agatha and Strüder‐Kypke 2013). Beyond the absence/presence of specific kineties and fields, some minor differences, such as the anterior elongation of the ventral kinety together with the last lateral kinety, the position of the posterior kinety, and/or the number of dikinetids at the beginning of the first and second right ciliary rows are promising distinguishing features at genus level. Yet, our current knowledge is too fragmentary to employ them (Agatha and Strüder‐Kypke 2007).

*Tintinnopsis campanula* is a supposed synonym of *T. bütschlii* (see above). Its original description by Ehrenberg (1840) is insufficient owing to the lack of an illustration. The redescription by Claparède and Lachmann (1858) is regarded as authoritative here, although that by Fauré‐Fremiet (1924) is more detailed, also considering cell features. In both *T. campanula* and *T. everta*, the cells project distinctly beyond the rims of the conspicuously flared collars, but the species differ clearly in their lorica lengths (150–200 μm vs. 61–115 μm; Claparède and Lachmann 1858), the opening diameter (about 84–109 μm as inferred from the original illustration vs. 46–88 μm) and the position of the right and left ciliary fields as indicated by their elongated anteriormost cilia (directly below vs. separated by a 7–11 μm broad, unciliated stripe from the membranellar zone; Fauré‐Fremiet 1924). Therefore, synonymy can be excluded. Hofker (1931) assumed a considerable variability in the lorica of *T. campanula* and proposed several further synonyms, but did not include *T. everta*. Hofker (1922, 1931) as well as Bakker and Phaff (1976) emphasised that these supposed variations all co‐occur with the typical *campanula* form, which has, however, not been found in our samples. The specimens from the Narragansett and Buzzards Bays at the east coast of the USA described by Pierce (1996) in his unpublished Doctoral Thesis and identified as *T. campanula* are actually conspecific with *T. everta*; this is supported by the lorica size and shape and, especially by the unique position of the posterior kinety.

Evidence for an identical kinetid structure in the posterior and dorsal kineties cumulated in previous studies. No other ciliary row in tintinnids is composed of dikinetids having a cilium associated only with each posterior basal body (Agatha and Strüder‐Kypke 2007), except for the posterior portion of the ventral kinety in *Schmidingerella arcuata*,* Tintinnopsis cylindrica*, and *T. tocantinensis* (Agatha and Strüder‐Kypke 2012; Coats et al. 2010; Jiang et al. 2012). Hence, homology of this posterior portion with the posterior kinety was discussed, but finally rejected; for arguments see Agatha and Strüder‐Kypke (2012). The occurrence of the posterior kinety seemed somehow to be correlated with the disappearance of the second dorsal kinety; only an undetermined “*Favella*” species has two dorsal kineties plus a posterior ciliary row [inferred from an illustration in Lynn and Small (2002)], and *Tintinnopsis brasiliensis* has only one dorsal kinety, but no posterior row (Cai et al. 2006). However, support for the hypothesised homology of the posterior kinety and the second (left) dorsal kinety beyond the identical kinetid structure was as yet absent. *Tintinnopsis everta* seems to represent this “missing link” that demonstrates the origin of the posterior kinety from the left dorsal kinety that shortened anteriorly and curved leftwards, diverging from the rightward bent dorsal kinety (Fig. 1C, 6B, S4, S6).

The evolution of the ventral kinety can also be reconstructed based on the known somatic ciliary patterns (Fig. S5, Table S2) and the opisthe's morphogenesis in *T. everta*, in which the anterior elongation and curving is apparently recapitulated (Fig. 9C). Simultaneously and parallel to the ventral kinety, the last kinety of the lateral ciliary field curves rightwards and gradually elongates anteriorly. All *Tintinnopsis* species, in which the ciliary pattern is known, as well as *Stenosemella pacifica* (Agatha and Tsai 2008) and *Rhizodomus tagatzi* (Saccà et al. 2012) demonstrate this feature, but to a greater or lesser extent. Only in *Stenosemella lacustris* (Foissner and O'Donoghue 1990; possibly, the junior synonym of *Tintinnopsis kiangsuensis*) both kineties are not elongated anteriorly. Even species without the most complex ciliary pattern, like *Eutintinnus angustatus* and *E. tenuis* (Choi et al. 1992), show tendencies of a joint curvature.

Bundles of argyrophilic fibres associated with the elongated collar membranelles and the buccal membranelle as well as with the dorsal portion of the adoral ring are not only found in *Tintinnopsis everta*, but have also been reported in *T. cylindrica* (Agatha and Riedel‐Lorjé 2006; Jiang et al. 2012), *T. fimbriata* (Agatha 2008), *T. tocantinensis* (Jiang et al. 2012), and *Stenosemella pacifica* (Agatha and Tsai 2008). In contrast to *T. everta*, however, the fibre bundles originating from the membranelles remain separate in these species.

Concerning the infection, we conclude that the parasite is an endoparasitic dinoflagellate most probably belonging to the genus *Euduboscquella* owing to a nuclear morphology closely resembling that of *E. crenulata* (Coats et al. 2012). While other dinoflagellate endoparasites infecting tintinnids have a single large nucleolus until late infection stages, the parasite of *T. everta* has many small nucleoli. A determination on species level is, however, impossible due to insufficient information on parasitic dinoflagellates in tintinnids.

### Phylogeny

Currently, the topology of the tintinnid genealogy is not settled. Although individual clades are generally well supported (> 90% probability in all four analyses), their relationships are unresolved owing to usually low support values (< 75% probability in at least three of the four analyses). Independent of the consideration of *T. beroidea* in the analyses, *T. everta* was occasionally affiliated with tintinnid clade 1, i.e. *T. major*,* T. bütschlii*, and *T. dadayi* (Fig. S1, S2). This grouping suggests that the lorica with a flared collar shared by these four species represents a synapomorphy. While this is probably true for *T. major*,* T. bütschlii*, and *T. dadayi* (genetic distances 0.3–3.6%), the genetic distances between *T. everta* and those species are, however, much larger (7.7–8.1%). Since the species of tintinnid clade 1, specifically *T. beroidea*, as well as *T. everta*, all display long branches invariably of the algorithm applied, their clustering might merely be a long‐branch‐attraction artefact.

Although the classification of the *Tintinnopsis*‐like tintinnids cannot be revised at present state of knowledge, it's so far unique complex somatic ciliary pattern and its large genetic distance to other *Tintinnopsis* species justify the separation of *T. everta* at genus level.

### Ontogenetic comparison

The cell division pattern of *T. everta* matches previous anecdotal observations in tintinnids with complex somatic ciliary patterns.

### Occurrence and ecology

Estimates of biogeography and autecology in *T. everta* are hampered owing to its potential confusion with *T. bütschlii*,* T. dadayi*, or *T. major* and unverifiable identification of some records. Actually, there are only two supported records besides this study; all of them are from the North Atlantic and adjacent sea areas. They belong to the cool‐temperate biogeographic zone: (i) the original type locality in the Kiel Bight, Germany, Baltic Sea, in September 1905 (Laackmann 1908) at a water temperature of about 15 °C and a salinity of about 17‰ [according to Lohmann (1908)] and (ii) the Narragansett and Buzzards Bay in July to October at salinities of 28–31 psu and water temperatures of 15–28 °C with abundances of up to 500 ind/L [incorrectly reported as *T. campanula*; Pierce (1996), see above]. Apparently, the species is eurythermal (15–30 °C) and occurs in meso‐ to polyhaline (15–30‰) coastal waters during summer.

The following records are unsubstantiated: New Caledonia, southwest Pacific Ocean (Dolan et al. 2006); near Pal‐Mi‐Island, Korea, northwest Pacific (Xu et al. 2000); Lebanese coastal waters, Mediterranean Sea (Abboud‐Abi Saab 2008); and east Skagerrak/Kattegat, Baltic Sea (Persson 2001).

## Taxonomic summary

Class Oligotrichea Bütschli, 1889

Order Choreotrichida Small and Lynn, 1985

Suborder Tintinnina Kofoid and Campbell, 1929

Genus *Tintinnopsis* Stein, 1867

***Tintinnopsis everta*** **Kofoid and Campbell,** **1929**

**Table:** 

1908	*Tintinnopsis baltica* var. *rotundata*—Laackmann, Wiss. Meeresunters., Abt. Kiel 10: 20, Plate 1, Fig. 9.
1929	*Tintinnopsis everta*—Kofoid and Campbell, Univ. Calif. Publ. Zool., 34: 35, Fig. 83.

### Remarks

As a diagnosis was not provided in the original description, the distinguishing features based on the type and neotype populations are presented here.

##### Neotype locality

The species was discovered in the Kiel Bight, Baltic Sea, and is here neotypified from the Chesapeake Bay, Maryland, at the east coast of the USA (37°44′N, 76°11′W).

##### Neotype material

Two slides (one neotype and one paratype slide) with protargol‐stained cells, including the neotype, further specimens, and the illustrated dividers, are deposited with the relevant cells marked in the Biology Centre of the Museum of Upper Austria (LI) in A‐4040 Linz (Austria). A neotype is established to provide stability in tintinnid taxonomy as (i) no type material is available, (ii) the original description lacks many morphologic and morphometric features, (iii) the species limits are unknown, and (iv) the genus is not monophyletic. For a detailed discussion of neotypification in ciliates, see Foissner (2002), Foissner et al. (2002), and Corliss (2003). The neotype is from the same cool‐temperate biogeographic zone as the original type locality (Laackmann 1908). Owing to the cosmopolitan distribution of the majority of marine planktonic ciliates, the establishment of a neotype from a site different from the type locality seems justified.

##### Diagnosis

Lorica on average 80 μm long, with an opening diameter of usually 50–80 μm; campanulate, composed of subspherical bowl and funnel‐shaped collar with on average four annuli. Bowl on average 40–50 × 40–55 μm. Lorica opening usually wider than bowl. Cell proper in vivo about 60 × 30 μm, obconical, after protargol staining on average 32 μm across. Two macronucleus nodules, two micronuclei. Somatic ciliary pattern of most complex type. Ventral kinety commences usually anteriorly to third right kinety. Right ciliary field with on average 11 kineties, distances between first four kineties conspicuously enlarged, first row without anterior dikinetid. Left ciliary field with on average ten kineties, lateral ciliary field with about 18 kineties. Distance between ciliary fields and collar membranelles extraordinarily large. Dorsal kinety composed of on average 27 dikinetids. Posterior kinety right of left field, composed of on average 15 dikinetids. About 19 collar membranelles, of which four extend into buccal cavity; one buccal membranelle.

## Supporting information


**Table S1.** GenBank accession numbers of SSU rRNA gene sequences of oligotrichid and choreotrichid species phylogenetically analysed in this study.
**Table S2.** Evolution of posterior and ventral kineties in tintinnids. The posterior kinety probably originated from the anteriorly shortened left dorsal kinety and curved successively leftwards below the left (LF) or lateral ciliary field (LA), while the ventral kinety elongated anteriorly to various degrees and curved rightwards above the right ciliary field (RF).
**Figure S1.** One of 54 best maximum parsimony trees of selected tintinnid species based on small subunit (SSU) rRNA gene sequences and computed with PAUP*. The numbers at the nodes represent the bootstrap values. Numbering of the tintinnid clades follows Santoferrara et al. (2017). The GenBank accession numbers are listed in the supplementary Table S1. *^1^ *Dadayiella ganymedes* had probably been confused with *D. bulbosa*; *^2^should possibly be affiliated with genus *Cyttarocylis* (Dolan et al. 2014);* **^3^according to Foissner et al. (1999) a synonym of *Codonella cratera*; *^4^possibly the senior synonym of *Stenosemella lacustris*; *^5^the sequenced specimen was misidentified as suggested by Agatha and Strüder‐Kypke (2014) and confirmed by Santoferrara et al. (2017), it is probably conspecific with *Dadayiella acutiformis* Kofoid and Campbell, 1939.
**Figure S2.** Small subunit (SSU) rRNA consensus tree of selected tintinnid species computed with MrBayes and based on the GTR + I + Γ model. The numbers at the nodes represent the posterior probability values. The scale bar represents 3 substitutions per 100 nucleotides. Numbering of the tintinnid clades follows Santoferrara et al. (2017). The GenBank accession numbers are listed in the supplementary Table S1. *^1^ *Dadayiella ganymedes* had probably been confused with *D. bulbosa*; *^2^should possibly be affiliated with genus *Cyttarocylis* (Dolan et al. 2014);* **^3^according to Foissner et al. (1999) a synonym of *Codonella cratera*; *^4^possibly the senior synonym of *Stenosemella lacustris*; *^5^the sequenced specimen was misidentified as suggested by Agatha and Strüder‐Kypke (2014) and confirmed by Santoferrara et al. (2017), it is probably conspecific with *Dadayiella acutiformis* Campbell, 1939.
**Figure S3.** Genetic distance tree of selected tintinnid species based on small subunit (SSU) rRNA gene sequences and computed with the Neighbor Joining algorithm in PHYLIP. The numbers at the nodes represent the bootstrap values. Numbering of the tintinnid clades follows Santoferrara et al. (2017). The scale bar represents 1 substitution per 100 nucleotides. The GenBank accession numbers are listed in the supplementary Table S1. *^1^ *Dadayiella ganymedes* had probably been confused with *D. bulbosa*; *^2^should possibly be affiliated with genus *Cyttarocylis* (Dolan et al. 2014);* **^3^according to Foissner et al. (1999) a synonym of *Codonella cratera*; *^4^possibly the senior synonym of *Stenosemella lacustris*; *^5^the sequenced specimen was misidentified as suggested by Agatha and Strüder‐Kypke (2014) and confirmed by Santoferrara et al. (2017), it is probably conspecific with *Dadayiella acutiformis* Kofoid and Campbell, 1939.
**Figure S4.** Schematic illustration showing the hypothesised evolution of the posterior kinety. In the ancestor, two dorsal kineties extended from the membranellar zone to the posterior end of cell proper. The left kinety shortened anteriorly (dashed line) and curved leftwards to various degrees (coloured lines). In contrast to this scheme, the increase in curvature actually did not cause a distinct elongation of the posterior kinety because of the obconical posterior portion of cell proper. The species with the particular pattern are listed (this study; Agatha 2008, 2010; Agatha and Tsai 2008; Jiang et al. 2012; Kim et al. 2010; inferred from illustrations in Lynn and Small 2002; Petz and Foissner 1993; Petz et al. 1995; Saccà et al. 2012); *possibly a junior synonym of *Tintinnopsis kiangsuensis*.
**Figure S5.** Schematic illustration showing the evolution of the ventral kinety. The ancestral pattern is represented by a longitudinal ventral kinety commencing at the same level as the remaining ciliary rows. Later, the row successively elongated anteriorly (colour‐coded) with the maximum extension in *Rhizodomus tagatzi* (Saccà et al. 2012). This evolution is recapitulated during morphogenesis of the opisthe in *T. everta* (cp. Fig. 10C). The species with the particular patterns are listed (this study; Agatha 2008, 2010; Agatha and Riedel‐Lorjé 2006; Agatha and Strüder‐Kypke 2012; Agatha and Tsai 2008; Cai et al. 2006; Choi et al. 1992; Foissner and O'Donoghue 1990; Foissner and Wilbert 1979; Jiang et al. 2012; Kim et al. 2010; Lynn and Small 2002; Petz et al. 1995; Sniezek et al. 1991; Snyder and Brownlee 1991); *possibly a junior synonym of *Tintinnopsis kiangsuensis*.
**Figure S6.** Kinetal maps showing the somatic ciliary patterns of morphostatic specimens in *Tintinnopsis everta* (A), *T. fimbriata* (B), *Rhizodomus tagatzi* (C), and *T. parvula* (D). Note that the posterior kineties (marked orange) extend longitudinally from their (anterior) starting points. Accordingly, their leftward shifting recognisable here corresponds to an increasing leftward curvature of the ciliary row because its posterior portion runs always parallel to the dorsal kinety for a certain distance (this study; Agatha 2008, 2010; Saccà et al. 2012).
**Figure S7.** Compilation of species with loricae similar to that of *Tintinnopsis everta*:* T. bacoorensis* (A; from Roxas 1941), *T. bütschlii* (B; from Daday 1887), *T. compressa* (C; from Daday 1887), *T. dadayi* (D; from Kofoid 1905), *T. directa* (E; from Hada 1932), *T. major* (F; from Meunier 1910), *T. manilensis* (G; from Roxas 1941), *T. mortensenii* (H; from Schmidt 1901), *T. orientalis* (I; from Kofoid and Campbell 1929), *T. patula* (J; from Meunier 1910), and *T. schotti* (K; from Brandt 1906). Scale bar about 50 μm.
