# Supplementary material for: Redescription of Tintinnopsis everta Kofoid and Campbell 1929 (Alveolata, Ciliophora, Tintinnina) Based on Taxonomic and Genetic Analyses—Discovery of a New Complex Ciliary Pattern
Source: J Eukaryot Microbiol. 2018 Jan 31;65(4):484–504. doi: 10.1111/jeu.12496 (PMC6055699; doi:10.1111/jeu.12496)
Supplement: Supplementary file 1 — Table S1. GenBank accession numbers of SSU rRNA gene sequences of oligotrichid and choreotrichid species phylogenetically analysed in this study. Table S2. Evolution of posterior and ventral kineties in tintinnids. The posterior kinety probably originated from the anteriorly shortened left dorsal kinety and curved successively leftwards below the left (LF) or lateral ciliary field (LA), while the ventral kinety elongated anteriorly to various degrees and curved rightwards above the right ciliary field (RF). Figure S1. One of 54 best maximum parsimony trees of selected tintinnid species based on small subunit (SSU) rRNA gene sequences and computed with PAUP*. The numbers at the nodes represent the bootstrap values. Numbering of the tintinnid clades follows Santoferrara et al. (2017). The GenBank accession numbers are listed in the supplementary Table S1. *1 Dadayiella ganymedes had probably been confused with D. bulbosa; *2should possibly be affiliated with genus Cyttarocylis (Dolan et al. 2014); *3according to Foissner et al. (1999) a synonym of Codonella cratera; *4possibly the senior synonym of Stenosemella lacustris; *5the sequenced specimen was misidentified as suggested by Agatha and Strüder‐Kypke (2014) and confirmed by Santoferrara et al. (2017), it is probably conspecific with Dadayiella acutiformis Kofoid and Campbell, 1939. Figure S2. Small subunit (SSU) rRNA consensus tree of selected tintinnid species computed with MrBayes and based on the GTR + I + Γ model. The numbers at the nodes represent the posterior probability values. The scale bar represents 3 substitutions per 100 nucleotides. Numbering of the tintinnid clades follows Santoferrara et al. (2017). The GenBank accession numbers are listed in the supplementary Table S1. *1 Dadayiella ganymedes had probably been confused with D. bulbosa; *2should possibly be affiliated with genus Cyttarocylis (Dolan et al. 2014); *3according to Foissner et al. (1999) a synonym of Codonella cratera; *4possibly the se [file JEU-65-484-s001.pdf]

SUPPORTING INFORMATION

Redescription of *Tintinnopsis everta* Kofoid & Campbell, 1929 (Alveolata, Ciliophora, Tintinnina) Based on Taxonomic and Genetic Analyses – Discovery of a New Complex Ciliary Pattern by Michael S. Gruber, Michaela Strüder-Kypke, Sabine Agatha

Figure S1 One of 54 best maximum parsimony trees of selected tintinnid species based on small subunit (SSU) rRNA gene sequences and computed with PAUP*. The numbers at the nodes represent the bootstrap values. Numbering of the tintinnid clades follows Santoferrara et al. (2017). The GenBank accession numbers are listed in the supplementary Table S1. *1 *Dadayiella ganymedes* had probably been confused with *D. bulbosa*; *2 should possibly be affiliated with genus *Cyttarocylis* (Dolan et al. 2014); *3according to Foissner et al. (1999) a synonym of *Codonella cratera*; *4possibly the senior synonym of *Stenosemella lacustris*; *5 the sequenced specimen was misidentified as suggested by Agatha and Strüder-Kypke (2014) and confirmed by Santoferrara et al. (2017), it is probably conspecific with *Dadayiella acutiformis* Kofoid and Campbell, 1939.

Figure S2 Small subunit (SSU) rRNA consensus tree of selected tintinnid species computed with MrBayes and based on the GTR+I+Γ model. The numbers at the nodes represent the posterior probability values. The scale bar represents 3 substitutions per 100 nucleotides. Numbering of the tintinnid clades follows Santoferrara et al. (2017). The GenBank accession numbers are listed in the supplementary Table S1. *1 *Dadayiella ganymedes* had probably been confused with *D. bulbosa*; *2 should possibly be affiliated with genus *Cyttarocylis* (Dolan et al. 2014); *3 according to Foissner et al. (1999) a synonym of *Codonella cratera*; *4 possibly the senior synonym of *Stenosemella lacustris*; *5 the sequenced specimen was misidentified as suggested by Agatha and Strüder-Kypke (2014) and confirmed by Santoferrara et al. (2017), it is probably conspecific with *Dadayiella acutiformis* Kofoid and Campbell, 1939.

Figure S3 Genetic distance tree of selected tintinnid species based on small subunit (SSU) rRNA gene sequences and computed with the Neighbor Joining algorithm in PHYLIP. The numbers at the nodes represent the bootstrap values. Numbering of the tintinnid clades follows Santoferrara et al. (2017). The scale bar represents 1 substitution per 100 nucleotides. The GenBank accession numbers are listed in the supplementary Table S1. *1 *Dadayiella ganymedes* had probably been confused with *D. bulbosa*; *2 should possibly be affiliated with genus *Cyttarocylis* (Dolan et al. 2014); *3according to Foissner et al. (1999) a synonym of *Codonella cratera*; *4possibly the senior synonym of *Stenosemella lacustris*; *5the sequenced specimen was misidentified as suggested by Agatha and Strüder-Kypke (2014) and confirmed by Santoferrara et al. (2017), it is probably conspecific with *Dadayiella acutiformis* Kofoid and Campbell, 1939.

Figure S4 Schematic illustration showing the hypothesised evolution of the posterior kinety. In the ancestor, two dorsal kineties extended from the membranellar zone to the posterior end of cell proper. The left kinety shortened anteriorly (dashed line) and curved leftwards to various degrees (coloured lines). In contrast to this scheme, the increase in curvature actually did not cause a distinct elongation of the posterior kinety because of the obconical posterior portion of cell proper. The species with the particular pattern are listed (this study; Agatha 2008, 2010; Agatha and Tsai 2008; Jiang et al. 2012; Kim et al. 2010; inferred from illustrations in Lynn and

Small 2002; Petz and Foissner 1993; Petz et al. 1995; Saccà et al. 2012); * possibly a junior synonym of *Tintinnopsis kiangsuensis*.

Figure S5 Schematic illustration showing the evolution of the ventral kinety. The ancestral pattern is represented by a longitudinal ventral kinety commencing at the same level as the remaining ciliary rows. Later, the row successively elongated anteriorly (colour-coded) with the maximum extension in *Rhizodomus tagatzi* (Saccà et al. 2012). This evolution is recapitulated during morphogenesis of the opisthe in *T. everta* (cp. Fig. 10C). The species with the particular patterns are listed (this study; Agatha 2008, 2010; Agatha and Riedel-Lorjé 2006; Agatha and Strüder-Kypke 2012; Agatha and Tsai 2008; Cai et al. 2006; Choi et al. 1992; Foissner and O'Donoghue 1990; Foissner and Wilbert 1979; Jiang et al. 2012; Kim et al. 2010; Lynn and Small 2002; Petz et al. 1995; Sniezek et al. 1991; Snyder and Brownlee 1991); * possibly a junior synonym of *Tintinnopsis kiangsuensis*.

Figure S6 Kinetal maps showing the somatic ciliary patterns of morphostatic specimens in *Tintinnopsis everta* (A), *T. fimbriata* (B), *Rhizodomus tagatzi* (C), and *T. parvula* (D). Note that the posterior kineties (marked orange) extend longitudinally from their (anterior) starting points. Accordingly, its leftward shifting recognisable here corresponds to an increasing leftward curvature of the ciliary row because its posterior portion runs always parallel to the dorsal kinety for a certain distance (this study; Agatha 2008, 2010; Saccà et al. 2012).

Figure S7 Compilation of species with loricae similar to that of *Tintinnopsis everta*: *T. bacoorensis* (A; from Roxas 1941), *T. bütschlii* (B; from Daday 1887), *T. compressa* (C; from Daday 1887), *T. dadayi* (D; from Kofoid 1905), *T. directa* (E; from Hada 1932), *T. major* (F; from Meunier 1910), *T. manilensis* (G; from Roxas 1941), *T. mortensenii* (H; from Schmidt 1901), *T. orientalis* (I; from Kofoid and Campbell 1929), *T. patula* (J; from Meunier 1910), and *T. schotti* (K; from Brandt 1906). Scale bar about 50 µm.

Table S1 GenBank accession numbers of SSU rRNA gene sequences of oligotrichid and choreotrichid species phylogenetically analysed in the present study.

Table S2. Evolution of posterior and ventral kineties in tintinnids. The posterior kinety probably originated from the anteriorly shortened left dorsal kinety and curved successively leftwards below the left (LF) or lateral ciliary field (LA), while the ventral kinety elongated anteriorly to various degrees and curved rightwards above the right ciliary field (RF).

SUPPLEMENTARY LITERATURE CITED

Agatha, S. 2008. Redescription of the tintinnid ciliate *Tintinnopsis fimbriata* Meunier, 1919 (Spirotricha, Choreotrichida) from coastal waters of Northern Germany. *Denisia,* 23:261-272.

Agatha, S. 2010. Redescription of *Tintinnopsis parvula* Jörgensen, 1912 (Ciliophora: Spirotrichea: Tintinnina), including a novel lorica matrix. *Acta Protozool.,* 49:213-234.

Agatha, S. & Riedel-Lorjé, J. C. 2006. Redescription of *Tintinnopsis cylindrica* Daday, 1887 (Ciliophora: Spirotricha) and unification of tintinnid terminology. *Acta Protozool.,* 45:137-151.

Agatha, S. & Strüder-Kypke, M. C. 2012. Reconciling cladistic and genetic analyses in choreotrichid ciliates (Ciliophora, Spirotricha, Oligotrichea). *J. Eukaryot. Microbiol.,* 59:325-350.

Agatha, S. & Strüder-Kypke, M. C. 2014. What morphology and molecules tell us about the evolution of Oligotrichea (Alveolata, Ciliophora). *Acta Protozool.,* 53:77-90.

Agatha, S. & Tsai, S.-F. 2008. Redescription of the tintinnid *Stenosemella pacifica* Kofoid and Campbell, 1929 (Ciliophora, Spirotricha) based on live observation, protargol impregnation, and scanning electron microscopy. *J. Eukaryot. Microbiol.,* 55:75-85.

Brandt, K. 1906. Die Tintinnodeen der Plankton-Expedition. Tafelerklärungen nebst kurzer Diagnose der neuen Arten. *Ergebn. Plankton-Exped. Humboldt-Stiftung,* 3 La:1-33 + Plates I-LXX.

Cai, S., Song, W., Xu, D. & Chiang, K. 2006. Morphological studies on the infraciliature of a planktonic ciliate, *Tintinnopsis brasiliensis* (Ciliophora: Tintinina). *J. Ocean Univ. China,* 5:55-57.

Chiang, S. C. 1956. Notes on the freshwater Tintinnoinea from Kiangsu and Anhui provinces. *Acta Hydrobiol. sin.,* 1:61-87.

Choi, J. K., Coats, D. W., Brownlee, D. C. & Small, E. B. 1992. Morphology and infraciliature of three species of *Eutintinnus* (Ciliophora; Tintinnina) with guidelines for interpreting protargol-stained tintinnine ciliates. *J. Protozool.,* 39:80-92.

Daday, E. v. 1887. Monographie der Familie der Tintinnodeen. *Mitt. zool. Stn Neapel,* 7:473-591 + Plates XVIII-XXI.

Dolan, J. R., Pierce, R. W. & Bachy, C. 2014. *Cyttarocylis ampulla*, a polymorphic tintinnid ciliate of the marine plankton. *Protist,* 165:66-80.

Foissner, W. & O'Donoghue, P. J. 1990. Morphology and infraciliature of some freshwater ciliates (Protozoa: Ciliophora) from Western and South Australia. *Invertebr. Taxon.,* 3:661-696.

Foissner, W. & Wilbert, N. 1979. Morphologie, Infraciliatur und Ökologie der limnischen Tintinnina: *Tintinnidium fluviatile* Stein, *Tintinnidium pusillum* Entz, *Tintinnopsis cylindrata* Daday und *Codonella cratera* (Leidy) (Ciliophora, Polyhymenophora). *J. Protozool.,* 26:90-103.

Foissner, W., Berger, H. & Schaumburg, J. 1999. Identification and Ecology of Limnetic Plankton Ciliates. Informationsberichte des Bayer. Landesamtes für Wasserwirtschaft 3/99. Bayer. Landesamt für Wasserwirtschaft, Munich. p. 793.

Hada, Y. 1932. Report of the biological survey of Mutsu Bay. 24. The pelagic ciliat, suborder Tintinnoinea. *Sci. Rep. Tohoku Imp. Univ., Ser. 4, Biol.,* 7:553-573.

Jiang, Y., Yang, J., Al-Farraj, S. A., Warren, A. & Lin, X. 2012. Redescriptions of three tintinnid ciliates, *Tintinnopsis tocantinensis*, *T. radix*, and *T. cylindrica* (Ciliophora, Spirotrichea), from coastal waters off China. *Eur. J. Protistol.,* 48:314-325.

Kim, S. Y., Yang, E. J., Gong, J. & Choi, J. K. 2010. Redescription of *Favella ehrenbergii* (Claparède and Lachmann, 1858) Jörgensen, 1924 (Ciliophora: Choreotrichia), with phylogenetic analyses based on small subunit rRNA gene sequences. *J. Eukaryot. Microbiol.,* 57:460-467.

Kofoid, C. A. 1905. Some new Tintinnidae from the plankton of the San Diego Region. *Univ. Calif. Publs Zool.,* 1:287-307.

Kofoid, C. A. & Campbell, A. S. 1929. A conspectus of the marine and fresh-water Ciliata belonging to the suborder Tintinnoinea, with descriptions of new species principally from the Agassiz Expedition to the eastern tropical Pacific 1904-1905. *Univ. Calif. Publs Zool.,* 34:1-403.

Kofoid, C. A. & Campbell, A. S. 1939. Reports on the scientific results of the expedition to the eastern tropical Pacific, in charge of Alexander Agassiz, by the U. S. Fish Commission Steamer "Albatross," from October, 1904, to March, 1905, Lieut.-Commander L. M. Garrett, U. S. N. Commanding. XXXVII. The Ciliata: The Tintinnoinea. *Bull. Mus. comp. Zool. Harv.,* 84:1-473 + Plates I-XXXVI.

Lynn, D. H. & Small, E. B. 2002. Phylum Ciliophora Doflein, 1901. *In*: Lee, J. J., Leedale, G. F. & Bradbury, P. (ed.), An Illustrated Guide to the Protozoa. 2nd edition. Organisms Traditionally Referred to as Protozoa, or Newly Discovered Groups (year 2000).371-656.

Meunier, A. 1910. Microplankton des Mers de Barents et de Kara. Campagne Arctique de 1907. C. Bulens, Bruxelles. p. xviii + 355 + Plates I-XXXVI.

Petz, W. & Foissner, W. 1993. Morphogenesis in some freshwater tintinnids (Ciliophora, Oligotrichida). *Eur. J. Protistol.,* 29:106-120.

Petz, W., Song, W. & Wilbert, N. 1995. Taxonomy and ecology of the ciliate fauna (Protozoa, Ciliophora) in the endopagial and pelagial of the Weddell Sea, Antarctica. *Stapfia,* 40:1-223.

Roxas, H. A. 1941. Marine protozoa of the Philippines. *Philipp. J. Sci. Manila,* 74:91-139 + Plates X-XVII.

Saccà, A., Strüder-Kypke, M. C. & Lynn, D. H. 2012. Redescription of *Rhizodomus tagatzi* (Ciliophora: Spirotrichea: Tintinnida), based on morphology and small subunit ribosomal RNA gene sequence. *J. Eukaryot. Microbiol.,* 59:218-231.

Santoferrara, L. F., Alder, V. V. & McManus, G. B. 2017. Phylogeny, classification and diversity of Choreotrichia and Oligotrichia (Ciliophora, Spirotrichea). *Mol. Phylogenet. Evol.,* 112:12-22.

Schmidt, J. 1902. Some Tintinnodea from the Gulf of Siam. *Vidensk. Meddel. Naturh. For. Kjøbenhavn,* year 1901:183-190.

Sniezek, J. H., Capriulo, G. M., Small, E. B. & Russo, A. 1991. *Nolaclusilis hudsonicus* n. sp. (Nolaclusiliidae n. fam.) a bilaterally symmetrical tintinnine ciliate from the lower Hudson River estuary. *J. Protozool.,* 38:589-594.

Snyder, R. A. & Brownlee, D. C. 1991. *Nolaclusilis bicornis* n. g., n.sp. (Tintinnina: Tintinnidiidae): a tintinnine ciliate with novel lorica and cell morphology from the Chesapeake Bay estuary. *J. Protozool.,* 38:583-589.

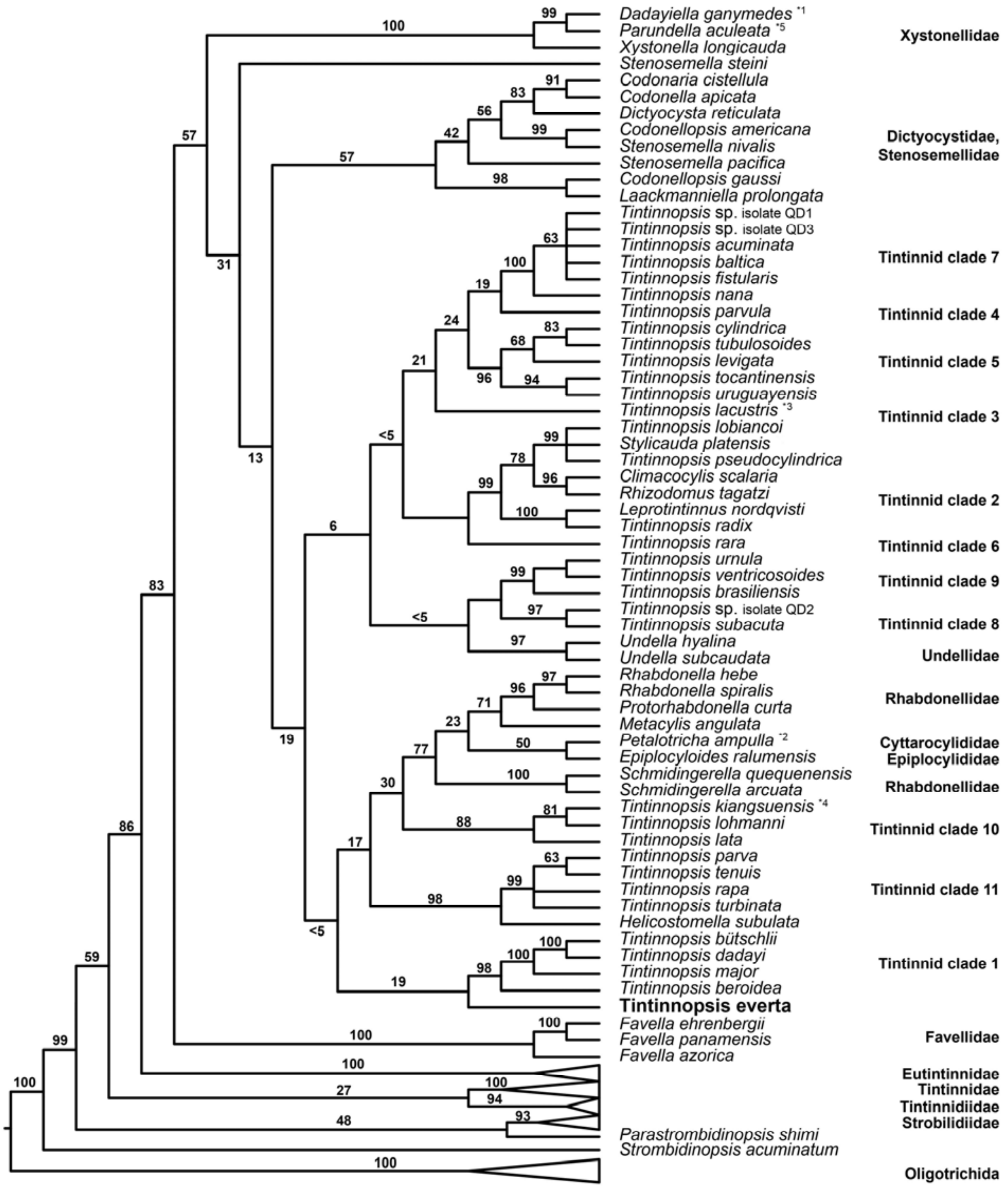
Dadayiella ganymedes *1
Parundella aculeata *5
Xystonella longicauda
Xystonellidae
Stenosemella steini
Codonaria cistellula
Codonella apicata
Dictyocysta reticulata
Codonellopsis americana
Stenosemella nivalis
Stenosemella pacifica
Dictyocystidae,
Stenosemellidae
Codonellopsis gaussi
Laackmanniella prolongata
Tintinnopsis sp. isolate QD1
Tintinnopsis sp. isolate QD3
Tintinnopsis acuminata
Tintinnopsis baltica
Tintinnid clade 7
Tintinnopsis fistularis
Tintinnopsis nana
Tintinnopsis parvula
Tintinnid clade 4
Tintinnopsis cylindrica
Tintinnopsis tubulosoides
Tintinnopsis levigata
Tintinnid clade 5
Tintinnopsis tocantinensis
Tintinnopsis uruguayensis
Tintinnopsis lacustris *3
Tintinnid clade 3
Tintinnopsis lobiancoi
Stylicauda platensis
Tintinnopsis pseudocylindrica
Climacocylis scalaria
Rhizodomus tagatzi
Leprotintinnus nordqvisti
Tintinnid clade 2
Tintinnopsis radix
Tintinnopsis rara
Tintinnid clade 6
Tintinnopsis urnula
Tintinnopsis ventricosoides
Tintinnid clade 9
Tintinnopsis brasiliensis
Tintinnopsis sp. isolate QD2
Tintinnopsis subacuta
Tintinnid clade 8
Undella hyalina
Undella subcaudata
Undellidae
Rhabdonella hebe
Rhabdonella spiralis
Protorhabdonella curta
Rhabdonellidae
Metacylis angulata
Petalotricha ampulla *2
Cyttarocylididae
Epiplocyloides ralumensis
Epiplocylididae
Schmidingerella quequenensis
Schmidingerella arcuata
Rhabdonellidae
Tintinnopsis kiangsuensis *4
Tintinnopsis lohmanni
Tintinnid clade 10
Tintinnopsis lata
Tintinnopsis parva
Tintinnopsis tenuis
Tintinnopsis rapa
Tintinnid clade 11
Tintinnopsis turbinata
Helicostomella subulata
Tintinnopsis bütschlii
Tintinnopsis dadayi
Tintinnopsis major
Tintinnid clade 1
Tintinnopsis beroidea
Tintinnopsis everta
Favella ehrenbergii
Favella panamensis
Favellidae
Favella azorica
Eutintinnidae
Tintinnidae
Tintinnidiidae
Strobilidiidae
Parastrombidinopsis shimi
Strombidinopsis acuminatum
Oligotrichida

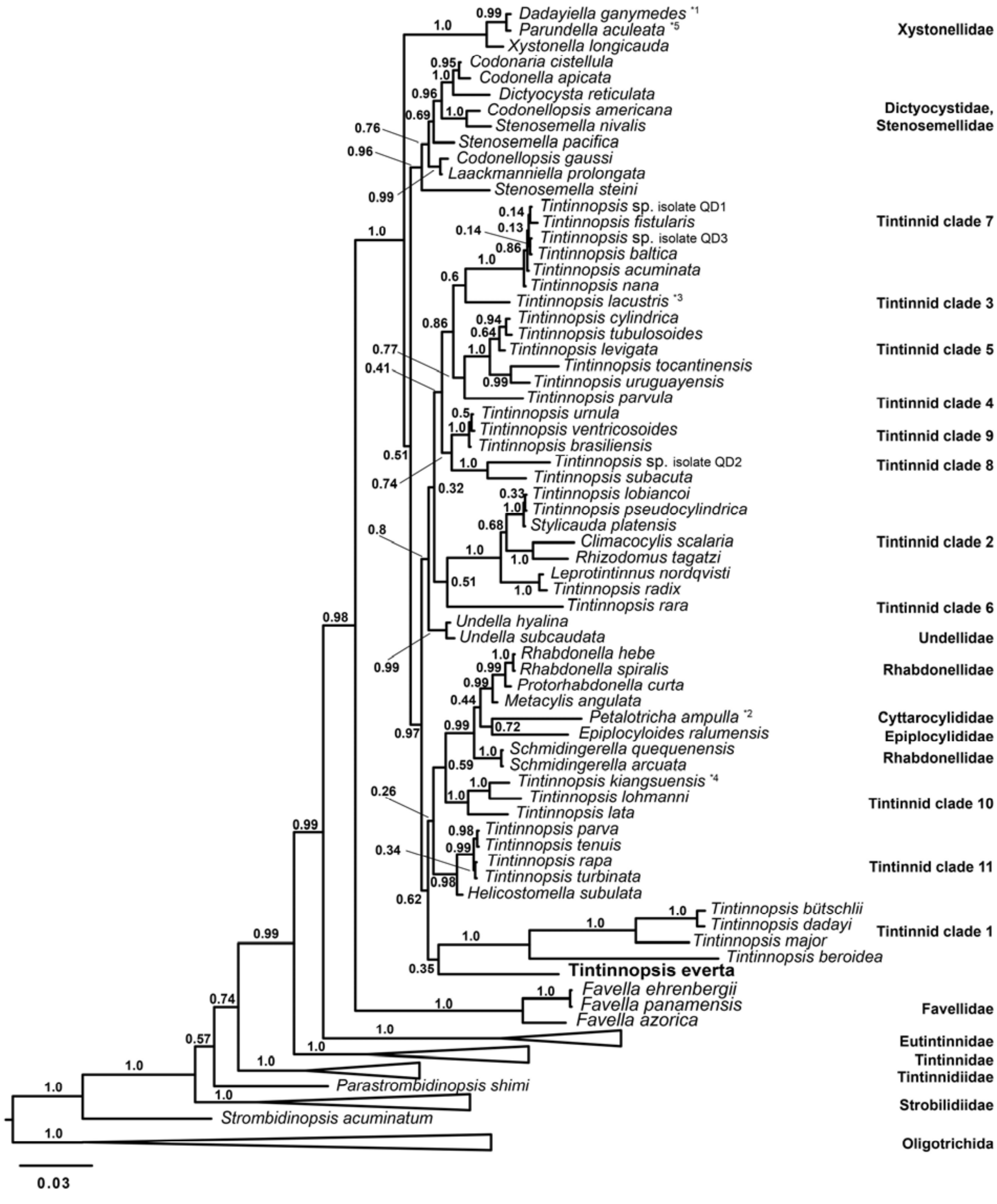
Dadayiella ganymedes *1
Parundella aculeata *5
Xystonella longicauda
Xystonellidae
Codonaria cistellula
Codonella apicata
Dictyocysta reticulata
Codonellopsis americana
Stenosemella nivalis
Dictyocystidae,
Stenosemellidae
Stenosemella pacifica
Codonellopsis gaussi
Laackmanniella prolongata
Stenosemella steini
Tintinnopsis sp. isolate QD1
Tintinnopsis fistularis
Tintinnopsis sp. isolate QD3
Tintinnid clade 7
Tintinnopsis baltica
Tintinnopsis acuminata
Tintinnopsis nana
Tintinnopsis lacustris *3
Tintinnid clade 3
Tintinnopsis cylindrica
Tintinnopsis tubulosoides
Tintinnopsis levigata
Tintinnid clade 5
Tintinnopsis tocantinensis
Tintinnopsis uruguayensis
Tintinnopsis parvula
Tintinnid clade 4
Tintinnopsis urnula
Tintinnopsis ventricosoides
Tintinnid clade 9
Tintinnopsis brasiliensis
Tintinnopsis sp. isolate QD2
Tintinnopsis subacuta
Tintinnid clade 8
Tintinnopsis lobiancoi
Tintinnopsis pseudocylindrica
Stylicauda platensis
Climacocylis scalaria
Rhizodomus tagatzi
Tintinnid clade 2
Leprotintinnus nordqvisti
Tintinnopsis radix
Tintinnopsis rara
Tintinnid clade 6
Undella hyalina
Undella subcaudata
Undellidae
Rhabdonella hebe
Rhabdonella spiralis
Rhabdonellidae
Protorhabdonella curta
Metacylis angulata
Petalotricha ampulla *2
Cyttarocylididae
Epiplocyloides ralumensis
Epiplocylididae
Schmidingerella quequenensis
Schmidingerella arcuata
Rhabdonellidae
Tintinnopsis kiangsuensis *4
Tintinnopsis lohmanni
Tintinnopsis lata
Tintinnid clade 10
Tintinnopsis parva
Tintinnopsis tenuis
Tintinnopsis rapa
Tintinnopsis turbinata
Tintinnid clade 11
Helicostomella subulata
Tintinnopsis bütschlii
Tintinnopsis dadayi
Tintinnopsis major
Tintinnid clade 1
Tintinnopsis beroidea
Tintinnopsis everta
Favella ehrenbergii
Favella panamensis
Favellidae
Favella azorica
Eutintinnidae
Tintinnidae
Tintinnidiidae
Parastrombidinopsis shimi
Strobilidiidae
Strombidinopsis acuminatum
Oligotrichida
0.03

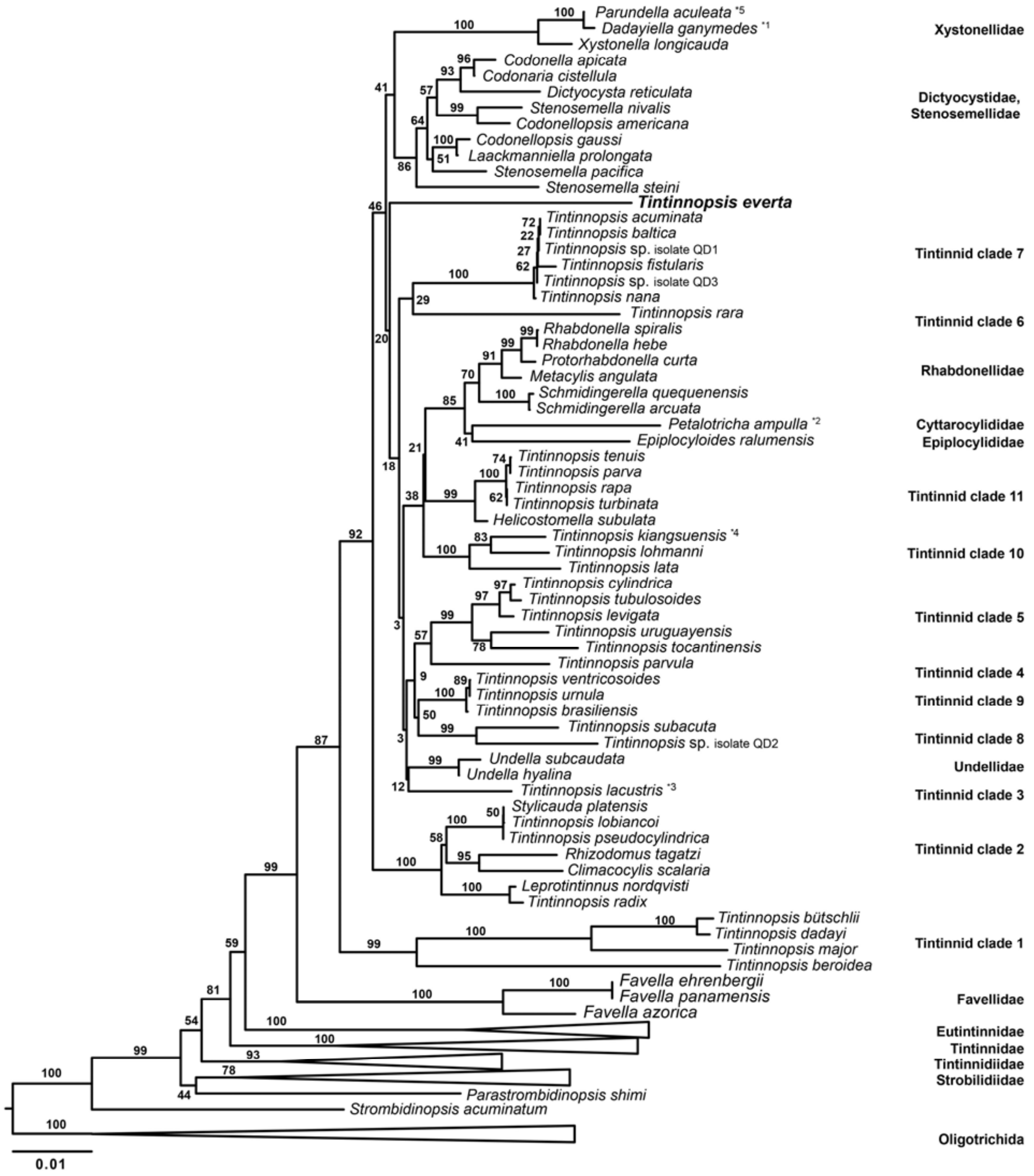
Parundella aculeata *5
Dadayiella ganymedes *1
Xystonella longicauda
Xystonellidae
Codonella apicata
Codonaria cistellula
Dictyocysta reticulata
Stenosemella nivalis
Codonellopsis americana
Codonellopsis gaussi
Laackmanniella prolongata
Stenosemella pacifica
Stenosemella steini
Dictyocystidae, Stenosemellidae
Tintinnopsis everta
Tintinnopsis acuminata
Tintinnopsis baltica
Tintinnopsis sp. isolate QD1
Tintinnopsis fistularis
Tintinnopsis sp. isolate QD3
Tintinnopsis nana
Tintinnid clade 7
Tintinnopsis rara
Tintinnid clade 6
Rhabdonella spiralis
Rhabdonella hebe
Protorhabdonella curta
Metacylis angulata
Schmidingerella quequenensis
Schmidingerella arcuata
Rhabdonellidae
Petalotricha ampulla *2
Cyttarocylididae
Epiplocyloides ralumensis
Epiplocylididae
Tintinnopsis tenuis
Tintinnopsis parva
Tintinnopsis rapa
Tintinnopsis turbinata
Helicostomella subulata
Tintinnid clade 11
Tintinnopsis kiangsuensis *4
Tintinnopsis lohmanni
Tintinnopsis lata
Tintinnid clade 10
Tintinnopsis cylindrica
Tintinnopsis tubulosoides
Tintinnopsis levigata
Tintinnopsis uruguayensis
Tintinnopsis tocantinensis
Tintinnid clade 5
Tintinnopsis parvula
Tintinnid clade 4
Tintinnopsis ventricosoides
Tintinnopsis urnula
Tintinnopsis brasiliensis
Tintinnid clade 9
Tintinnopsis subacuta
Tintinnopsis sp. isolate QD2
Tintinnid clade 8
Undella subcaudata
Undella hyalina
Undellidae
Tintinnopsis lacustris *3
Tintinnid clade 3
Stylicauda platensis
Tintinnopsis lobiancoi
Tintinnopsis pseudocylindrica
Rhizodomus tagatzi
Climacocylis scalaria
Leprotintinnus nordqvisti
Tintinnopsis radix
Tintinnid clade 2
Tintinnopsis bütschlii
Tintinnopsis dadayi
Tintinnopsis major
Tintinnopsis beroidea
Tintinnid clade 1
Favella ehrenbergii
Favella panamensis
Favella azorica
Favellidae
Eutintinnidae
Tintinnidae
Tintinnidiidae
Strobilidiidae
Parastrombidinopsis shimi
Strombidinopsis acuminatum
Oligotrichida
0.01

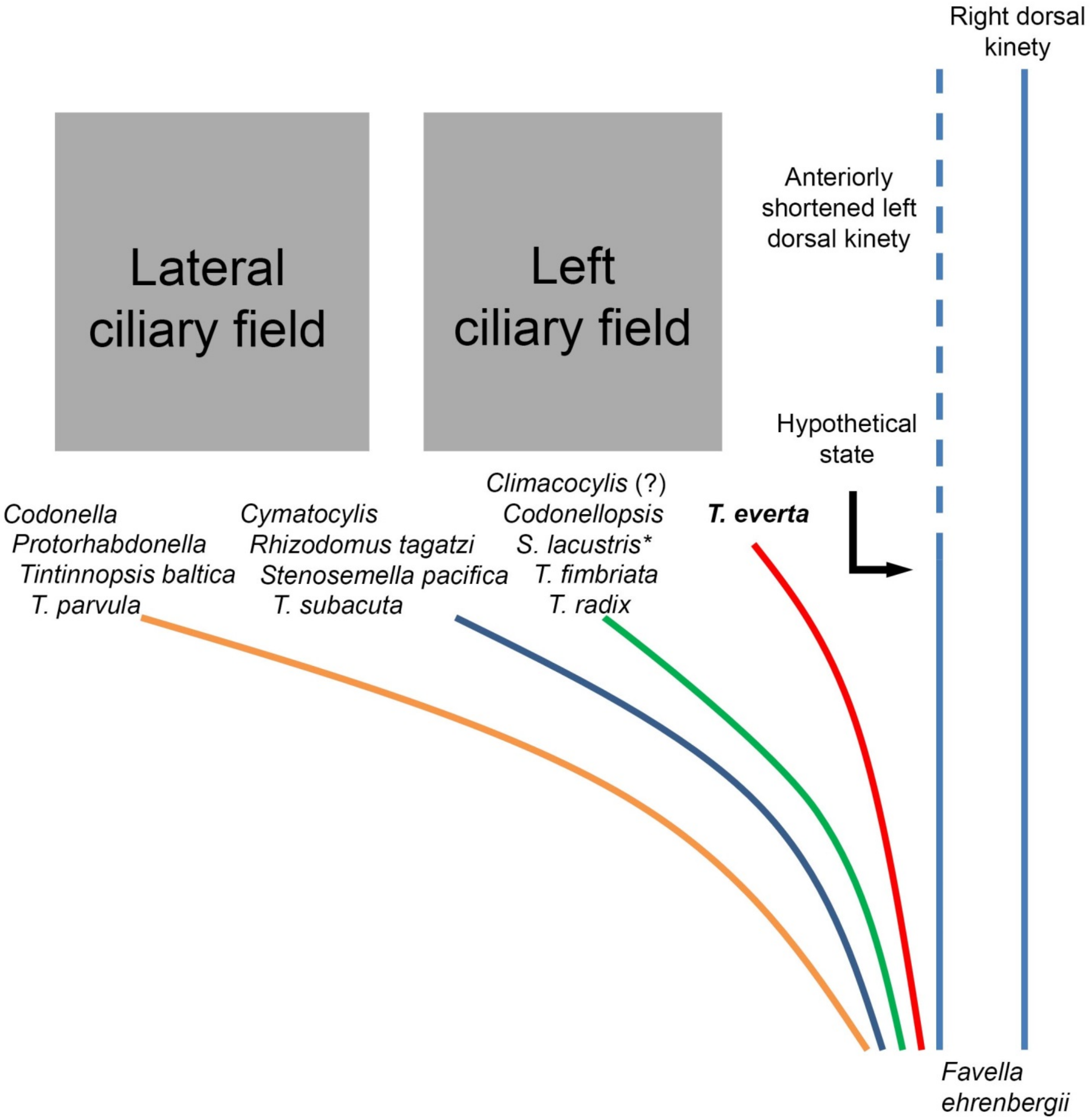

Right dorsal kinety
Lateral ciliary field
Left ciliary field
Anteriorly shortened left dorsal kinety
Hypothetical state
Codonella
Protorhabdonella
Tintinnopsis baltica
T. parvula
Cymatocylis
Rhizodomus tagatzi
Stenosemella pacifica
T. subacuta
Climacocylis (?)
Codonellopsis
S. lacustris*
T. fimbriata
T. radix
T. everta
Favella ehrenbergii

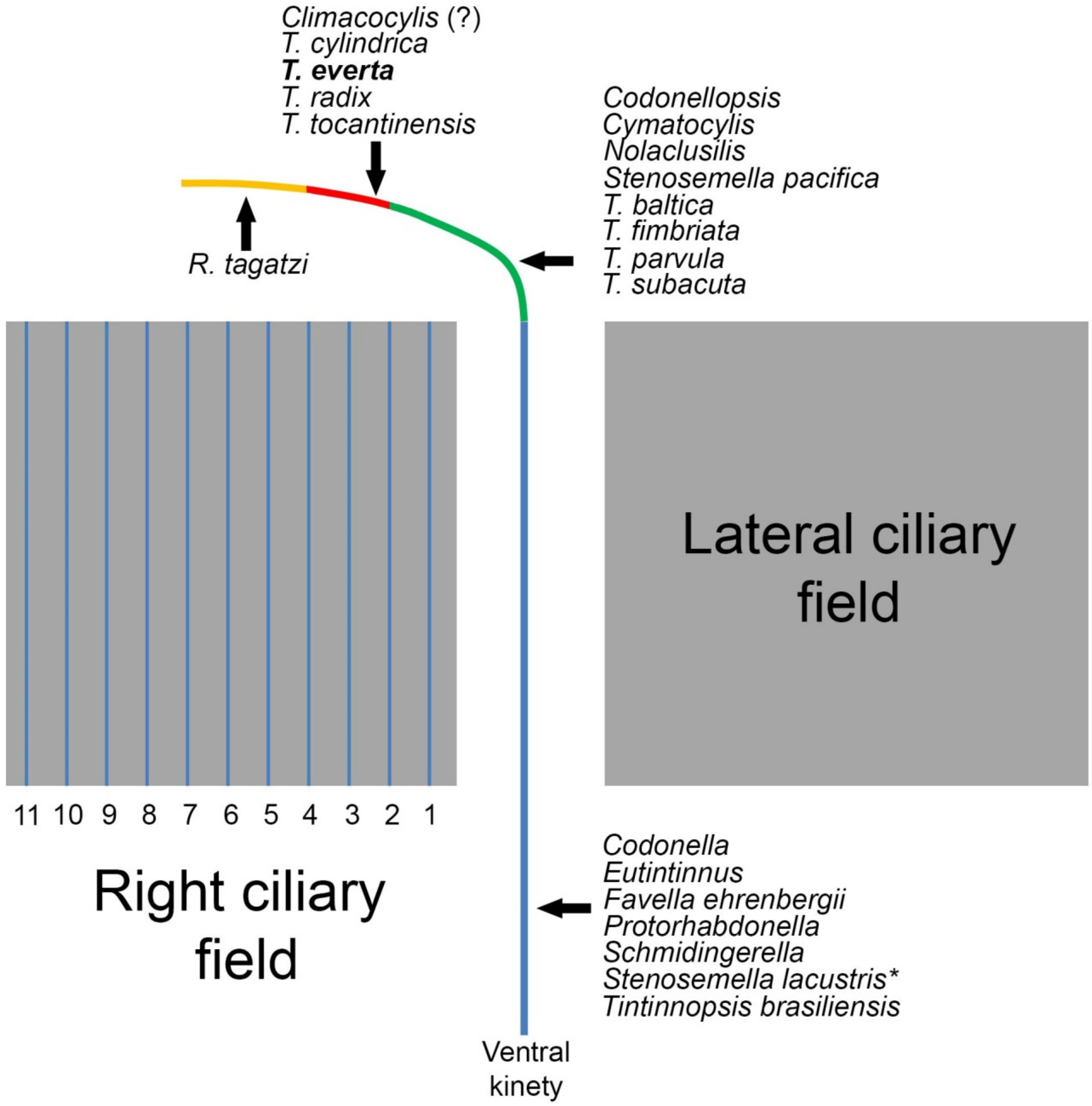
Climacocylis (?)
T. cylindrica
T. everta
T. radix
T. tocantinensis
Codonellopsis
Cymatocylis
Nolaclusilis
Stenosemella pacifica
T. baltica
T. fimbriata
T. parvula
T. subacuta
R. tagatzi
Lateral ciliary field
11 10 9 8 7 6 5 4 3 2 1
Right ciliary field
Codonella
Eutintinnus
Favella ehrenbergii
Protorhabdonella
Schmidingerella
Stenosemella lacustris*
Tintinnopsis brasiliensis
Ventral kinety

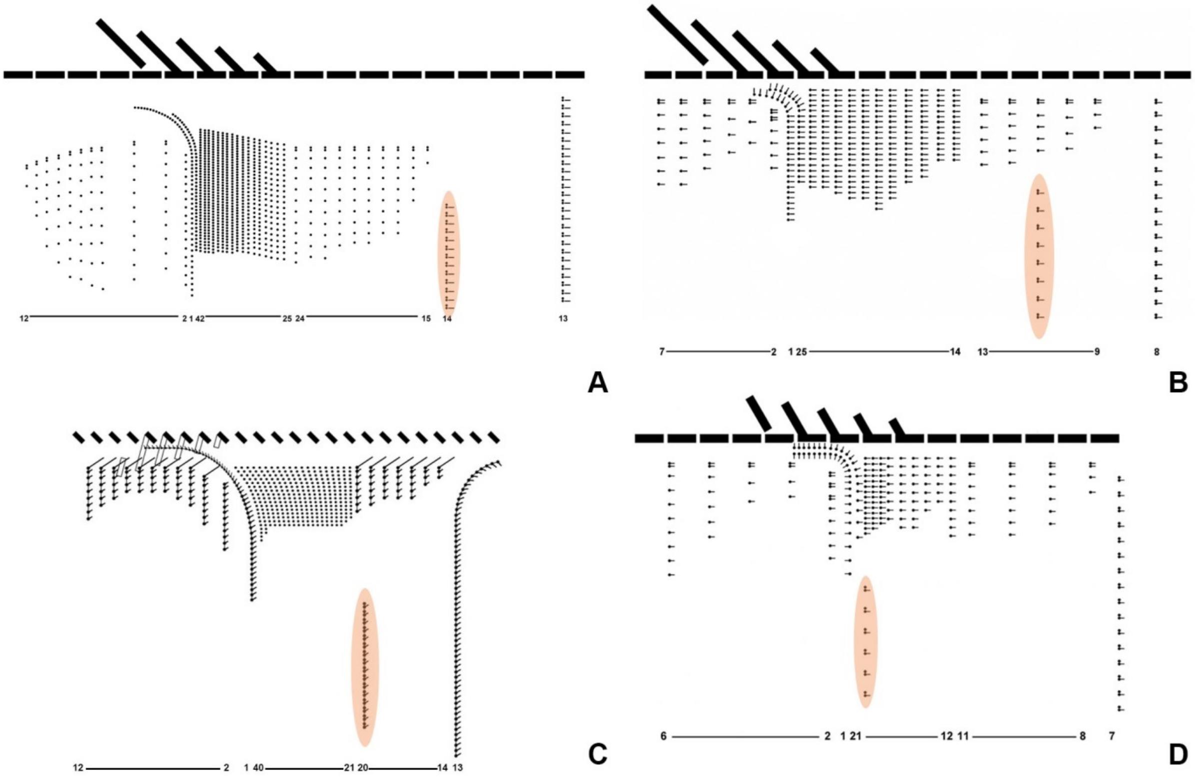
12
2 1 42
25 24
15
14
13
A
7
2
1 25
14
13
9
8
B
12
2
1 40
21 20
14 13
C
6
2
1 21
12 11
8
7
D

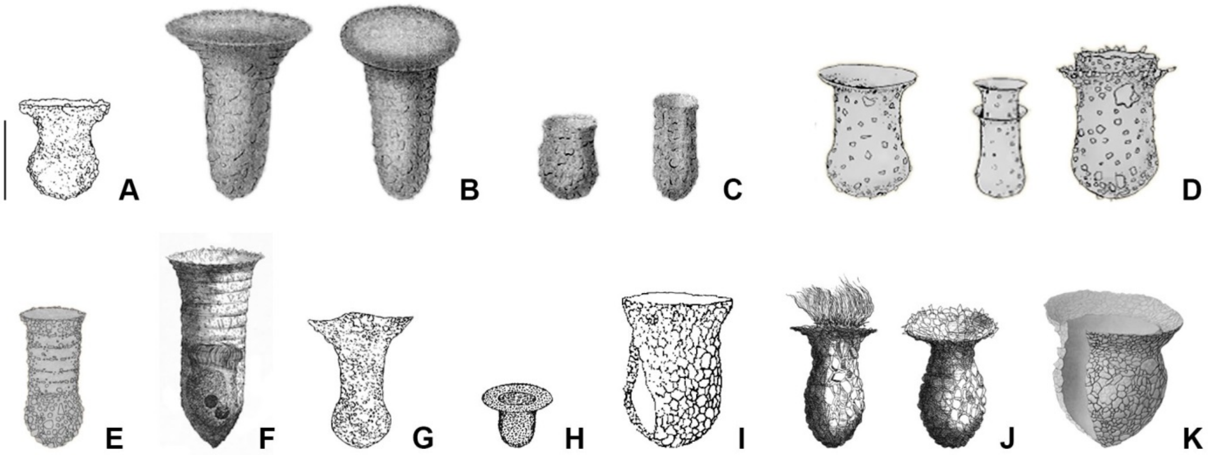
A
B
C
D
E
F
G
H
I
J
K

Table S1. GenBank accession numbers of SSU rDNA gene sequences of oligotrichid and choreotrichid species phylogenetically analysed

Full Name	GenBank Accession No
Amphorellopsis acuta	EU399530
Amphorides amphora	JX101849
Climacocylis scalaria	JQ408213
Codonaria cistellula	JQ408202
Codonella apicata	EU399531
Codonellopsis americana	AY143571
Codonellopsis gaussi	JQ924055
Dadayiella ganymedes (*1)	JX101852
Dictyocysta reticulata	EU399532
Epiplocyloides ralumensis	JX101854
Eutintinnus fraknoi	EU399534
Eutintinnus pectinis	AY143570
Eutintinnus tenuis	JN871721
Favella azorica	JQ408212
Favella ehrenbergii	GU574769
Favella panamensis	AY143572
Helicostomella subulata	JN831784
Laackmanniella prolongata	JQ924057
Laboea strobila	AY302563
Leprotintinnus nordqvisti	KU715761
Metacylis angulata	AY143568
Novistrombidium testaceum	AJ488910
Parallelostrombidium obesum	FJ422991
Parastrombidinopsis shimi	AJ786648
Parundella aculeata (*5)	JQ408204
Pelagostrobilidium neptuni	AY541683
Petalotricha ampulla (*2)	JQ408185
Protorhabdonella curta	JX101863
Pseudotontonia simplicidens	FJ422993
Rhabdonella hebe	AY143566
Rhabdonella spiralis	KT792932
Rhizodomus tagatzi	JQ392572
Rimostrombidium lacustris	DQ986131
Salpingella acuminata	JQ408155
Schmidingerella arcuata	JQ837815
Schmidingerella quequenensis	KU715765
Spirotontonia turbinata	FJ422994
Steenstrupiella steenstrupii	KT792924
Stenosemella nivalis	FJ196074
Stenosemella pacifica	JN831793
Stenosemella steini	KT792927
Strobilidium caudatum	AY143573
Strombidinopsis acuminata	AJ877014
Strombidium apolatum	DQ662848
Strombidium basimorphum	FJ480419
Strombidium biarmatum	AY541684

Stylicauda platensis	JN831832
Tintinnidium fluviatile	JQ408163
Tintinnidium mucicola	AY143563
Tintinnopsis acuminata	JN831844
Tintinnopsis baltica	JN831805
Tintinnopsis beroidea	EF123709
Tintinnopsis brasiliensis	KU715768
Tintinnopsis bütschlii	JN831810
Tintinnopsis cylindrica	FJ196075
Tintinnopsis dadayi	AY143562
Tintinnopsis everta	MG461220
Tintinnopsis fistularis	KU715770
Tintinnopsis kiangsuensis (*4)	JN831850
Tintinnopsis lacustris (*3)	JQ408161
Tintinnopsis lata	KM982810
Tintinnopsis levigata	KM982811
Tintinnopsis lobiancoi	JN831814
Tintinnopsis lohmanni	FJ196076
Tintinnopsis major	JN831816
Tintinnopsis nana	JN831821
Tintinnopsis parva	JN831824
Tintinnopsis parvula	JN831830
Tintinnopsis pseudocylindrica	JN831855
Tintinnopsis radix	EU399540
Tintinnopsis rapa	JN831834
Tintinnopsis rara	JQ408200
Tintinnopsis sp.	FJ422985
Tintinnopsis sp.	FJ422986
Tintinnopsis sp.	FJ422987
Tintinnopsis subacuta	EU399541
Tintinnopsis tenuis	JN831848
Tintinnopsis tocantinensis	AY143561
Tintinnopsis tubulosoides	AF399108
Tintinnopsis turbinata	JN831846
Tintinnopsis urnula	JN831852
Tintinnopsis uruguayensis	EU399542
Tintinnopsis ventricosoides	KU715776
Undella hyalina	JQ408207
Undella subcaudata	KT792931
Xystonella longicauda	JQ408211

[*1] *Dadayiella ganymedes* had probably been confused with *D. bulbosa*; [*2] should possibly be affiliated with genus *Cyttarocylis* (Dolan et al., 2014); [*3] according to Foissner et al. (1999) a synonym of *Codonella cratera*; [*4] possibly the senior synonym of *Stenosemella lacustris*; [*5] the sequenced specimen was misidentified as suggested by Agatha and Strüder-Kypke (2014) and confirmed by Santoferrara et al. (2017), it is probably conspecific with *Dadayiella acutiformis* Kofoid And Campbell, 1939.

Table S2. Evolution of posterior and ventral kineties in tintinnids. The posterior kinety probably originated from the anteriorly shortened left dorsal kinety and curved successively leftwards below the left (LF) or lateral ciliary field (LA), while the ventral kinety elongated anteriorly to various degrees and curved rightwards above the right ciliary field (RF).

**Anterior portion of posterior kinety**			**Anterior portion of ventral kinety**				
Right of LF	Underneath LF	Underneath LA	Longitudinal (plesiomorphic)	Above 1st kinety of RF	Above 2nd kinety of RF	Above 3rd kinety of RF	Above >3rd kinety of RF
Tintinnopsis everta [17]	*Climacocylis* [6, 12]	*Codonella* [9]	*Codonella* [9]	*Codonellopsis* [13]	*Nolaclusilis* [15, 16]	*T. everta* [17]	*Climacocylis* [6]
	Codonellopsis [13]	*Protorhabdonella* [12]	*Eutintinnus* [7]	*Cymatocylis* [13]	*Stenosemella pacifica* [5]	*T. tocantinensis* [10]	*Rhizodomus* [14]
	Cymatocylis [13]	*T. baltica* [12]	*Favella* [11]		*Tintinnopsis baltica* [12]		*T. cylindrica* [3, 10]
	Rhizodomus [14]	*T. parvula* [2]	*Protorhabdonella* [12]		*T. fimbriata* [1]		*T. everta* [17]
	Stenosemella lacustris [8]		*Schmidingerella* [4]		*T. parvula* [2]		*T. radix* [10]
	T. fimbriata [1]		*Stenosemella lacustris* [8]		*T. subacuta* [12]		*T. tocantinensis* [10]
	T. radix [10]						
	T. subacuta [12]						

[1] Agatha (2008); [2] Agatha (2010); [3] Agatha and Riedel-Lorjé (2006); [4] Agatha and Strüder-Kypke (2012); [5] Agatha and Tsai (2008); [6] Brownlee (1977); [7] Choi et al. (1992); [8] Foissner and O'Donoghue (1990); [9] Foissner and Wilbert (1979); [10] Jiang et al. (2012); [11] Kim et al. (2010); [12] Lynn and Small (2002); [13] Petz et al. (1995); [14] Saccà et al. (2012); [15] Sniezek et al. (1991); [16] Snyder and Brownlee (1991); [17] present study.
